# Diagnostic prediction models for bacterial meningitis in children with a suspected central nervous system infection: a systematic review and prospective validation study

**DOI:** 10.1136/bmjopen-2023-081172

**Published:** 2024-08-07

**Authors:** Nina S Groeneveld, Merijn W Bijlsma, Ingeborg E van Zeggeren, Steven L Staal, Michael W T Tanck, Diederik van de Beek, Matthijs C Brouwer

**Affiliations:** 1 Department of Neurology, Amsterdam UMC Location AMC, Amsterdam, The Netherlands; 2 Department of Pediatrics, Amsterdam UMC Location AMC, Amsterdam, The Netherlands; 3 Department of Epidemiology and Data Science, Amsterdam UMC—Locatie AMC, Amsterdam, The Netherlands

**Keywords:** neurology, infectious diseases, diagnostic microbiology, decision making

## Abstract

**Abstract:**

**Objectives:**

Diagnostic prediction models exist to assess the probability of bacterial meningitis (BM) in paediatric patients with suspected meningitis. To evaluate the diagnostic accuracy of these models in a broad population of children suspected of a central nervous system (CNS) infection, we performed external validation.

**Methods:**

We performed a systematic literature review in Medline to identify articles on the development, refinement or validation of a prediction model for BM, and validated these models in a prospective cohort of children aged 0–18 years old suspected of a CNS infection.

**Primary and secondary outcome measures:**

We calculated sensitivity, specificity, predictive values, the area under the receiver operating characteristic curve (AUC) and evaluated calibration of the models for diagnosis of BM.

**Results:**

In total, 23 prediction models were validated in a cohort of 450 patients suspected of a CNS infection included between 2012 and 2015. In 75 patients (17%), the final diagnosis was a CNS infection including 30 with BM (7%). AUCs ranged from 0.69 to 0.94 (median 0.83, interquartile range [IQR] 0.79–0.87) overall, from 0.74 to 0.96 (median 0.89, IQR 0.82–0.92) in children aged ≥28 days and from 0.58 to 0.91 (median 0.79, IQR 0.75–0.82) in neonates.

**Conclusions:**

Prediction models show good to excellent test characteristics for excluding BM in children and can be of help in the diagnostic workup of paediatric patients with a suspected CNS infection, but cannot replace a thorough history, physical examination and ancillary testing.

**STRENGTHS AND LIMITATIONS OF THIS STUDY**

- A strength of our study is that we present an extensive overview of published prediction models that predict the likelihood of bacterial meningitis. We furthermore validated these models in data from the Paediatric and Adult Causes of Encephalitis and Meningitis study. This was a multicentre prospective study in three hospitals in which patients aged 0–18 years old with a suspected CNS infection in which cerebrospinal fluid (CSF) examination was performed, were included.
- Another strength of this study is that we evaluated the performance of these prediction models by assessing both discrimination and calibration.
- A limitation of this study is that the median number of missing values per variable was 12% (IQR 4%–42%). Missing data were handled by multiple imputation.

## Introduction

 Bacterial meningitis (BM) in children is lethal and debilitating, with mortality rates between 4% and 21% and neurological sequelae occurring in up to one-third of survivors.^1^,^3^ Early start of treatment is crucial for the prognosis as delay in antibiotic treatment is associated with adverse outcomes.^4^ However, limiting unnecessary use of antibiotics is important to minimise antibiotic resistance, adverse reactions, hospital admissions and healthcare costs.^5^

Recognition of BM can be difficult. The typical triad of fever, neck stiffness and altered mental status is present in only 41% of adult patients and is even less common in children and infants.^6 7^ Diagnostic prediction models have been developed to help identify which child should be treated for BM and in which a watchful waiting approach can be applied.^8^ The majority of these models combine clinical and laboratory findings and predict the probability of acute BM, compared with viral meningitis or no meningitis. However, substantial differences between these models exist, especially with respect to patient populations and diagnostic criteria. Validation of prediction models in a broader population of patients suspected of a central nervous system (CNS) infection is necessary because this is the population in which these models will be of clinical use, however is often lacking. External validation of 16 diagnostic prediction models for BM in a cohort of 363 adult patients with a suspected CNS infection showed that none of the existing models performed well enough to recommend routine use in individual patient management. However, these models were mostly developed for children and might therefore perform better in a paediatric population.

Our aim was to perform a systematic review of prediction models for BM and validate these models using a multicentre cohort of paediatric patients with a suspected CNS infection in whom a lumbar puncture was performed.

## Methods

### Systematic review

We systematically reviewed the literature in Medline to identify models that predict the probability of acute BM. The Standards for Reporting Diagnostic accuracy studies 2015 guidelines and Transparent Reporting of a multivariable prediction model for Individual Prognosis Or Diagnosis: checklist for systematic reviews and meta-analyses (TRIPOD-SRMA) were applied.^9 10^ We used a previously validated search filter for prediction models.^11^ We combined this filter with terms for meningitis and prediction models and searched for full-text articles in scientific peer-reviewed journals from January 1980 to 1 September 2022 in languages English, German, French, Spanish or Dutch. Prediction models were included if they contained at least three variables obtained from history, physical examination or simple laboratory tests and included children or adults. Publications describing the development, refinement or validation of a prediction model were included. Article screening and data extraction were performed by one researcher (NSG) and doubts were discussed and resolved by a second and third researcher (MCB and MWB). Quality of the reporting of the included studies (that were not previously included) was assessed according to the TRIPOD criteria, containing 6 domains with in total 22 items.^12^ Each item was scored as reported, reported incompletely and not reported. Characteristics and model performance of studies previously described (published before August 2018) were reported in the online supplemental material, and studies included after August 2018 were reported in the main manuscript.^8^

### Validation cohort

Data from the Paediatric and Adult Causes of Encephalitis and Meningitis (PACEM) study were used for validation of the included prediction models. This was a multicentre prospective study in three hospitals (the Amsterdam University Medical Centers, Onze Lieve Vrouwe Gasthuis in Amsterdam and the Flevoziekenhuis in Almere) in which patients were included if (1) aged 0–18 years old, (2) presented to the emergency department or admitted to the paediatric ward between January 2012 and July 2015 with a suspected CNS infection and (3) cerebrospinal fluid (CSF) examination was performed.^13^ A detailed description of the cohort was described previously.^13^

Change in mental status was defined as a Paediatric Glasgow Coma Scale (GCS) <14; coma was defined as a GCS <8.^14^ Episodes were categorised into six categories regarding final diagnosis: BM, other CNS infections, inflammatory CNS diseases, systemic infections, other neurological diseases and other systemic disease.^15^ All episodes were independently assessed by two clinicians (NSG and SLS) and discrepancies were discussed and resolved by a third and fourth clinician (MCB and MWB). BM was defined as (1) a positive CSF culture, or (2) a negative CSF culture but positive blood culture and elevated CSF leucocyte count, or (3) a negative CSF and blood culture, elevated CSF leucocyte count, elevated infection parameters in blood (CRP >80 mg/L or leucocytes >14 × 10^9^ /L), clinical parameters suggesting a bacterial infection and the final diagnosis of the treating physician. Age-specific cut-off values for abnormal CSF leucocyte count, protein and glucose were used. In children below 3 months >9 leucocytes/mm^3^ was considered elevated; in children of 3 months or older >6 leucocytes/mm^3^ was used as cut-off.^16^,^18^ CSF protein >1000 mg/L and CSF glucose levels <60% of blood glucose levels were considered abnormal. CSF leucocyte count was corrected for CSF erythrocytes by subtracting one leucocyte for every 700 erythrocytes/mm^3^.

### Statistics

The differences in baseline characteristics between BM and non-BM patients were identified with parametric and non-parametric tests. χ^2^ tests and Fisher’s exact tests were used to compare categorical outcomes.

The performance of the prediction models was assessed by evaluating discrimination and calibration.^19^,^21^ The different prediction models were considered as index test; diagnosis of BM was considered reference standard. Discrimination was assessed by calculating the area under the receiver operating characteristic curves (AUC) with 95% confidence intervals (CIs). Calibration was evaluated with the calibration curve, assessing the calibration slope and calculating the calibration in the large. Discriminative ability was categorised as follows: excellent discrimination in case of an AUC of ≥0.90; good discrimination for 0.80≤ AUC <0.90; fair discrimination for 0.70≤ AUC <0.80; and poor discrimination in case of an AUC <0.70.^22^ When cut-off values for defining high risk of BM had been provided in the original article, sensitivity, specificity and predictive values for the high-risk categories were calculated.

For some of the multivariable logistic regression prediction models, beta coefficients from the original publication were not reported. In this case, we used the observed proportions in the risk categories as reported in the original cohort data, as expected proportions in those risk groups in the validation data.

In case of unclear or missing information about the specifics of the prediction model, authors were contacted for additional information.

In models that reported the complete multivariable logistic regression model, proportions of BM assigned to the different risk categories, defined by the original model, were calculated to display the clinical significance of this spread. A sensitivity analysis in neonates (age <28 days) and in children ≥28 days of age was performed, because of the different presentation of BM at the neonatal age.^23^

The median number of missing values per variable was 12% (IQR 4%–42%). Missing data were handled by multiple imputation using the R package MICE. We used 51 variables (Supplementary methods) from medical history, physical examination and laboratory results as predictors to impute missing values.^24^ Missing data were regarded as missing at random and different regression models were used depending on the type of variable: logistic regression for dichotomous variables, polytomous regression for unordered categorical variables with >2 levels and predictive mean matching for numerical variables. For skewed numerical variables and derived variables (e.g., age <7 days), the so-called passive imputation was applied with log or square-root transformation where appropriate. A total of 30 imputation sets were generated based on the observed percentage missingness. If only one predictor from the model was not available in the PACEM dataset, the prediction model was validated without that particular variable. When two or more predictors from the model were not available in our dataset, the model was not validated. If a continuous predictor was missing but the median or mean value (of the original cohort) of that predictor was reported in the original article, this mean/median value was set as observed value in the validation of the prediction model. For discrimination and calibration, we used R packages pROC 22^25^ and predictABEL.^26^

We used Rubin’s rule and bootstrapping to estimate proportions and c-statistics based on the imputation sets.^27^ All statistical tests were two-tailed and p values of <0.05 were considered statistically significant.

### Patient and public involvement

None.

## Results

### Systematic review

Our literature search yielded 7724 articles of which 40 publications on diagnostic prediction models for acute BM were included. In total, 29 publications described the derivation of a total of 32 prediction models and 8 articles described an update, validation or explanation of a model.^28^,^65^ In total, 20 articles on 23 prediction models were included for validation in our study (online supplemental figure S1). Thirteen publications validated one or more existing models in their dataset (tables1  2).^2831 34 43 45 47 49 54 56^,^60 66^ All models were based on clinical characteristics and/or laboratory test results from blood and CSF. Characteristics of the derivation cohorts and performance measures of original models published after August 2018 are presented in tables1  2, and models published before August 2018 were described in detail previously, with the exception of one study published in 2011 that was not described before (online supplemental tables S1 and S2).^8 45^ A total of 23 models were developed in children^29^,^3133^ of which 7 in neonates,^34 42 53 64 65^ with a median cohort size in the derivation studies of 398 (IQR 158–908) patients. Five models were developed in both adults and children^40 50 63 67^ and three in adults.^32 33 52^ The most frequent quality limitations were retrospective derivation cohorts, lack of reporting on handling of missing data and little information about differences in distribution of important variables between the derivation and validation cohort (online supplemental table S3).

**Table 1:** Characteristics of included prediction models (published after August 2018)

	Population	Modelling method	Items	Cut-off
Boum *et al*^31^	Children 2 months–12 years, history of fever and ≥1 sign of CNS involvement	LRM	Neck stiffness, positive Brudzinski or Kernig sign, bulging fontanel, CSF leucocyte count >100 cells/µL or peripheral neutrophils >10 000 cells/mm^3^.	BM likely if ≥1 items present
Chen *et al*^34^	Full-term neonates aged ≤28 days with sepsis and an LP	SRA	Fever, absent source of infection, neurological manifestation (seizure, abnormal muscle tone, irritability or bulging anterior fontanelle) CRP >25 mg/L and PCT >2.5 ng/mL, CRP >50 mg/L and PCT>30 ng/mL	High risk if presence of ≥1 items
Cheng *et al*^64^	Preterm infants with an LP	LR	0.1258× PCT level on first day after birth+1.2542 × prenatal glucocorticoid use (yes=1, no=0)–0.1089 × albumin level –0.2557×1 min Apgar –0.1838 × BiPAP days –0.0172 × haemoglobin level on first day after birth –0.8131 × sex (female=2, boy=1)	NR
Dalai *et al*^65^	Neonates with an LP	LRM	−6.42+0.59 if no apnoea+2.05 if no irritability+0.18 if no high-pitched cry+1.01 if no seizures+1.26 if no neutrophilia+0.66 if normal CRP – 0.002 × SNAPPE-II score+1.75 if no leucomalacia	BM unlikely if score 0
Huang *et al*^41^	Term neonates with an LP	LRM	Model 1: −8.47839+0.19469 × CSF WBC+0.00190 × CSF protein+0.01667 × CSF glucose – 0.00503×LDHModel 2 = −6.34939+0.14552 × CSF WBC+0.00069 × CSF protein – 0.05915×CSF glucose.	BM likely if score > −1.7032 BM likely if score > −2.8547
Li *et al*^42^	Full-term infants aged ≤28 days or premature infants <40 weeks PMA with an LP	SRA	Gender, birth weight, level of glucose in CSF, WBCs in CSF, hsCRP level in blood and LDH level in blood.*	NR
Mentis *et al*^63^	Patients of all ages with meningitis	ML	Group 1 (G1)=CSF neutrophils and CSF lymphocytes; group 2 (G2)=G1 + CSF NLR; group 3 (G3)=G2 + blood albumin; group 4 (G4)=G3 + gender + age; group 5 (G5)=G4 + blood glucose; group 6 (G6)=G5 + blood CRP; group 7 (G7)=G6 + blood soluble urokinase-type plasminogen activator receptor; group 8 (G8)=G7 + lymphocytes-to-blood CRP ratio. Cut-off CSF neutrophils=287 cells/µl, cut-off CSF NLR=2. Other cut-offs not reported.	NR
Mintegi *et al*^43^	Children 29 days–14 years with meningitis	LRM	Serum PCT >1.20 ng/mL = 3 points, CSF protein >80 mg/dL = 2 points, CSF ANC >1000/μL=1 point, serum CRP >40 mg/L = 1 point	BM likely if score ≥1
Mirkhani *et al*^44^	Patients suspected of meningitis	DTA	Rule 1: CSF WBC count >32, CSF protein level >51, CSF glucose level ≤50 Rule 2: CSF WBC>32, CSF protein level>73, CSF glucose level>50	BM likely if all items of rule 1 or 2 are present
Mwaniki *et al*^45^	Infants <90 days of age with meningitis	LRM	History of fever, history of convulsions, bulging fontanel, irritability, axillary temperature ≥39	BM likely if ≥1 item present
Pelkonen *et al*^49^	Infants <90 days of age with an LP	LRM	Age >7 days, weight <2500g, ill>7 days, seizures on admission, signs of shock or prolonged capillary refill and unclear CSF	BM likely if ≥1 items present
Wang *et al*^53^	Full-term neonates 0–28 days with CSF pleiocytosis	LRM	Peripheral blood ANC ≥10×10^9^ cells/L, CSF protein level ≥1650 mg/L, CSF ANC ≥84×10^6^ cells/L, a positive CSF gram stain and a history of seizure before or at the time of presentation.	Low risk if no item present

* Variables were not clearly specified for this model.

ANC, absolute neutrophil countBiPAP, biphasic positive airway pressure; BM, bacterial meningitis; BMS, Bacterial Meningitis Score; CNS, central nervous system; CRP, C reactive protein; CSF, cerebrospinal fluid; DTA, decision tree algorithm; hsCRP, high-sensitive C reactive protein; LDH, lactate dehydrogenase; LP, lumbar puncture; LR, Lasso regression; LRM, logistic regression model; ML, machine learning; NLR, neutrophil-to-lymphocyte ratio; NR, not reported; PCT, procalcitonin; PMA, post menstrual age; SNAPPE-II, score for acute neonatal physiology and perinatal extension II; SRA, stepwise logistic regression analysis; WBC, white blood cell count

**Table 2:** Derivation and previous validation of identified prediction models (published after August 2018)

	Population	Source of data	Sample size	Original model performance	Internal/external validation
Boum *et al*^31^	Children 2 months–12 years with a suspected CNS infection, Uganda, 2009–2012	Prospective database cohort, n=459	60 BM	Sens 93.3%, spec 64.4%	NA
Chen *et al*^34^	Full-term neonates aged ≤28 days with sepsis and an LP, China, 2010–2019	Prospective and retrospective database cohort, n=689	102 BM	Sens 96.2%, accuracy 98.9%	Chen *et al*, 2021^34^ (p, n=383): sens 95.9%, accuracy 98.9%
Cheng *et al*^64^	Preterm infants with an LP, China, 2017–2020	Prospective cohort, n=168	77 BM, 91 AM	Brier score: 0.17, c- slope:0.966, C-index: 0.82 (95% CI 0.75 to 0.89)	NA
Dalai *et al*^65^	Neonates with suspected sepsis and an LP, India, 2014–2016	Prospective cohort, n=300	121 definite/possible BM, 191 no BM	C-statistic: 0.67 (95% CI 0.60 to 0.73),P value <0.001. Accuracy: 66.2%.	NA
Huang *et al*^41^	Term neonates with an LP, China, 2000–2017	Retrospective database cohort, n=1830	105 BM, 1725 AM	1: Sens 95.1%, spec 99.7%, AUC 0.982: Sens 95.1%, spec 99.7%, AUC 0.98	NA
Li *et al*^42^	Full-term infants ≤28 days or premature <40 weeks PMA, with an LP, China, 2012–2018	Retrospective database cohort, n=997	236 BM	Sens 52.97%, spec 96.98%, AUC 0.91	NA
Mentis et al*^63^	Patients of all ages with CSF samples at the National Meningitis Reference Laboratory, Greece	Retrospective database cohort, n=4339 cases	1758 BM, 2581 VM	Median sens MLR : 63% (IQR 61%–65%)Median sens RF : 67% (IQR 67%–70%)Median sens NB : 60% (58%–63%)	NA
Mintegi *et al*^43^	Children 29 days–14 years with meningitis, Spain, 2011–2018	Retrospective and prospective database cohort, n=819	61 BM, 758 AM	Sens 100%, spec 83.2%	Mintegi *et al*, 2020^43^ (p, n=190)**:** Sens 100%, spec 77.4%, AUC=0.986, p<0.0001
Mirkhani *et al*^44^	Individualssuspected of meningitis, Iran, 2009–2011	Retrospective database cohort, n=7945	2219 BM, 2291 AM	Sens 87%, spec 70%, AUC 0.84	NA
Mwaniki *et al*^45^	All admissions aged <60 days with an LP, Kenya, 2001–2007	Prospective database cohort, n=3923	Age <7 days: 31 BMAge 7–59 days: 76 BM	Age <7 days: sens 84%, spec 67% Age 7–59 days: sens 94%, spec 30%	Obiero (Arch) (r, n=4809), sens: 79%, spec 51%;Mwaniki *et al,* 2011^45^ (p, n=1512): sens 77.8%, spec 55.2%; Pelkonen 2021 (p, n=568): sens 45%, spec 68%
Pelkonen *et al*^49^	Infants <90 days of age with BM or sepsis signs/symptoms with an LP, Angola, 2016–2017	Prospective database cohort, n=1088	212 confirmed BM, 88 probable BM	Sens 97%, spec 16%	NA
Wang *et al*^53^	Full-term neonates 0–28 days with CSF pleiocytosis, China, 2001–2019	Prospective and retrospective database cohort, n=475	94 BM (20%), 381 AM (80%)	Sens 100%, spec 70.9%	NA

* Only results from patients aged 0–14 years are shown here; medians of the different groups (G1–-G8, table 1) were calculated per statistical model.

AM, aseptic meningitis; AUC, area under the curve; BM, bacterial meningitis; C-index, concordance index; CNS, central nervous system; CSF, cerebrospinal fluid; c-slopecalibration-slopeLP, lumbar puncture; MLR, multiple logistic regression; NA, not applicable; NB, naïve-BayesPMA, postmenstrual age; p, prospective study; r, retrospective study; RF, random forest; sens, sensitivity; spec, specificity

### Description of cohort

Between 2012 and 2015, a total of 468 episodes were included, of which 450 episodes could be used in the analysis (table 3). Reasons for exclusion were lack of information in online and paper files (n=14), multiple admissions of one patient in a short timeframe (n=2) and age at admission of 18 years or older (n=2). Included patients were female in 194 out of 450 (43%) cases, and median age at admission was 1.5 months (IQR 0.4–12). A total of 75% of children were <1 year old, 40% of children were <28 days old and 92 of 176 (52%) of neonates were born prematurely. For the analyses, three cohorts were used: the entire cohort of all children (n=450), neonates only (<28 days of age, n=176) and children aged ≥28 days (n=274). The neonate cohort included 16 (9%) BM cases and the children aged ≥28 days included 14 (5%) BM cases.

**Table 3:** Baseline characteristics validation cohort*

	BM (n=30)	No BM (n=420)	P value
Female sex	11/30 (37%)	183/420 (44%)	0.57
Age, months	0.5 (0.1–13.5)	1.6 (0.5–12.1)	0.10
Prematurity	8/26 (31%)	84/334 (25%)	0.53
Symptoms <24 hours	15/29 (52%)	212/373 (57%)	0.59
AB started before LP	6/27 (22%)	28/393 (7%)	0.005
Otitis media	4/30 (13%)	24/371 (6%)	0.15
Sinusitis	4/30 (13%)	49/380 (13%)	0.78
Pneumonia	1/30 (3%)	4/386 (1%)	0.31
Endocarditis	0/30 (0%)	0/399 (0%)	>0.99
General symptoms			
Fever	17/27 (63%)	251/393 (64%)	>0.99
Irritability	16/16 (100%)	177/401 (44%)	<0.001
Vomiting	6/25 (24%)	81/371 (22%)	0.80
Diarrhoea	3/27 (11%)	48/377 (13%)	>0.99
Headache	3/21 (14%)	36/318 (11%)	0.72
Purpura/petechial rash	4/30 (13%)	10/420 (2%)	<0.001
Vital signs			
Heart rate (beats/min)†	161 (±43)	156 (±33)	0.448
Systolic BP (mm Hg)‡	93 (±21)	100 (±23)	0.307
Diastolic BP (mm Hg)‡	53 (±12)	58 (±19)	0.312
Neurological symptoms			
Seizures§	3/30 (10%)	48/420 (11%)	0.82
Focal	0/3 (0%)	6/48 (13%)	>0.99
Generalised	2/3 (67%)	19/48 (40%)	0.56
GCS <14	5/30 (17%)	43/420 (10%)	0.27
GCS <8	1/30 (3%)	20/420 (5%)	>0.99
Bulging fontanel	0/6 (0%)	6/43 (14%)	>0.99
Meningeal irritation	5/15 (33%)	43/234 (18%)	0.15
Focal deficits	1/24 (4%)	23/394 (6%)	0.73
Laboratory findings blood			
CRP (mg/L)¶	47 (5–155)	8 (2–28)	<0.001
Thrombocyte count (×10^9^/L)**	207 (142–289)	279 (211–373)	<0.05
Leucocyte count (×10^9^/L)††	16.6 (9.1–21.1)	9.8 (7.1–14.1)	<0.001
Laboratory findings CSF			
Leucocyte count (cells/mm^3^)‡‡	191 (54–2244	3 (1–6)	0.001
Protein level (g/L)§§	1.23 (0.6–2.1)	0.40 (0.22–0.74)	<0.001
CSF:blood glucose ratio¶¶	0.51 (±0.30)	0.67 (±0.26)	<0.006

Data are presented as n/N (%), median (IQR) or mean (±SD). Corrected for CSF erythrocyte count if possible, data on CSF leucocyte count available in 429 patients and data on CSF erythrocyte count available in 271 patients.

* Data are presented as no. of patients/no. of patients in which these data are available. Type of seizure unknown in

† Data available in 331 patients.Data available in , data available in , data available in , data available in , data available in , data available in , data available in .

‡ Data available in 123 patients.Corrected for CSF erythrocyte count if possible, data on CSF leukocyte count available in , data on CSF erythrocyte count available in .

§ Type of seizure unknown in 20/48 (42%) patients.

¶ Data available in 425 patients.

** Data available in 427 patients.

†† Data available in 431 patients.

‡‡ Corrected for CSF erythrocyte count if possible, data on CSF leucocyte count available in 429 patients, data on CSF erythrocyte count available in 271 patients.

§§ Data available in 421 patients.

¶¶ Data available in 265 patients.

AB, antibiotics; BM, bacterial meningitis; BP, blood pressure; CRP, C reactive protein; CSF, cerebrospinal fluid; GCS, Glasgow Coma Scale; LP, lumbar puncture

Symptoms were present <24 hours in 227 of 402 (56%) patients. Most common symptoms were fever in 268 of 420 (64%), irritability in 193 of 429 (45%), meningeal irritation in 48 of 249 (19%) and a decreased level of consciousness 80 in 450 (18%). Median CSF leucocyte count in all children was 4 (IQR 1–9). CSF examination showed elevated leucocytes (corrected for CSF erythrocyte count) in 70 of 258 (27%) patients below 3 months old and in 32 of 166 (19%) patients of 3 months old or older. CSF protein was elevated in 73 of 419 (17%) in all patients and CSF to blood glucose ratio was decreased in 104 of 263 (39%) in all patients.

CNS infection was diagnosed in 74 of 450 (16%) of patients, of which 30 (41%) had BM, 39 (53%) viral meningitis and 5 (7%) infectious encephalitis. Other diagnose categories included CNS inflammatory disease (3%), systemic infection (61%), other neurological disease (14%) and other systemic disease (6%). CSF culture was positive in 10 of 30 patients (30%) clinically diagnosed with BM and showed *Streptococcus pneumoniae* in 3, *S. agalactiae* in 3, *Neisseria meningitidis* in 2, *Haemophilus influenzae* in 1 and *Escherichia coli* in 1. Blood culture was positive in 11 of 30 BM patients (37%) and showed *S. agalactiae* in 4, *H. influenza* in 1, *S. pneumoniae* in 2, *K. pneumoniae* in 1, *N. meningitidis* in 2 and *E. coli* in 1. One patient had a positive CSF culture for *S. pneumonia* and a negative blood culture, one patient had a positive blood culture for *K. pneumoniae* and a negative CSF culture and in one patient CSF culture failed due to a traumatic lumbar puncture and blood culture was positive for *S. agalactiae*

### Validation of prediction models

We validated 23 prediction models in our cohort: discrimination and calibration could be calculated for 13 models and sensitivity, specificity, positive predictive value (PPV) and negative predictive value (NPV) could be calculated for 20 models. The models of Cheng, Chen, Dalai, Dubos, Freedman, Mintegi and Pelkonen were excluded for validation because these models included two or more predictors that were not available in our PACEM dataset.^34 38 39 43 49 64 65^ The model of Mentis and the model of Li were not validated because these model were not reported in sufficient detail to perform validation.^42 63^ In total, 36 (80%) of the total number of 45 predictors from the 25 models were available in our dataset (online supplemental table S4). For the model of Boum, we were unable to assign points for a positive Brudzinski or Kernig sign; however, since the variable neck stiffness was considered a good proxy, this model was validated in our cohort. For the model of Spanos, the logistic regression model was used to calculate the AUC and calibration, and the CSF predictors (Spanos criteria) were used to calculate sensitivity, specificity, NPV and PPV. For the model of Huang, the median of lactate dehydrogenase as observed in the original cohort was imputed to validate the model in our cohort; however, this led to a negligible difference in predictions when compared with leaving out this variable. For the model of Mintegi, three out of a total of seven points could be appointed for procalcitonin, but this value was not available in our dataset and the contribution of this variable to the total model was considered high. Therefore, this model was not validated by using observed values from the original cohort. For the model of Bonsu (2008) and de Cauwer, the predicted probabilities could not be calculated because the betacoefficients of the original modal could not be retrieved. However, observed proportions per risk intervals from the original cohort were available and were used as the expected proportion for validation.^30 35^ Finally, we could assign no more than two points for duration of symptoms in days for the model of Oostenbrink because these data were not available in our dataset.^48^ We adjusted the original cut-off for the model of Oostenbrink by reducing the maximum amount of points with the percentage of points that were not available due to the missing values. Moreover, predictive values were calculated for the combined Oostenbrink model only.

#### All children

Discrimination was excellent in 2 models in all children and good in 6 of 13 models (table 4). The AUCs in these models ranged from 0.69 to 0.94 (median 0.83, IQR 0.79–0.87). The models of Bonsu, Nigrovic, the clinical model of Oostenbrink and the LRM model of Spanos showed an AUC below 0.80, indicating fair discrimination. In all children, the second model of Huang scored best in terms of discrimination with an AUC of 0.94 (CI 0.91 to 0.97). Moreover, sensitivity of Huang was 83% (95% CI 80% to 87%), with 90% specificity (95% CI 87% to 93%), 37% PPV (95% CI 33% to 42%) and 99% NPV (95% CI 99% to 100%). The calibration slopes indicated poor fit of all the models and none of the calibration curves showed reasonable agreement between the predicted and observed probability. Only the model of Boyer and Nigrovic showed a p value of the calibration slope >0.05. Moreover, calibration-in-the-large showed overestimation or underestimation in all of the models (table 4, online supplemental figure S2). As an example, the proportions of BM in low-risk and high-risk groups are shown in online supplemental table S5.

**Table 4:** Discrimination and calibration for all children

	AUC (95% CI)	Calibration in the large (95% CI)	Calibration slope (95% CI), p value
Bonsu	0.75 (0.65 to 0.86)	34% (29% to 39%)	Slope: 0.06 (0.03–0.09), p<0.001
Bonsu 2	0.87 (0.79 to 0.95)	−5% (−8% to −2%)	Slope: 1.6 (1.1–2.0), p<0.001
Boyer	0.83 (0.72 to 0.94)	2% (−1% to 6%)	Slope: 0.9 (0.6–1.2), p=0.34
Chavanet—adults	0.88 (0.80 to 0.95)	NA	NA
Chavanet—children	0.82 (0.73 to 0.90)	NA	NA
De Cauwer	0.85 (0.78 to 0.91)	16% (12% to 21%)	Slope: 1.9 (1.3 to 2.6), p<0.001
Hoen	0.84 (0.76 to 0.91)	11% (7% to 15%)	Slope: 0.07 (0.04 to 0.11), p<0.001
Huang—model 1	0.94 (0.90 to 0.97)	NA	NA
Huang—model 2	0.94 (0.91 to 0.97)	NA	NA
Nigrovic	0.79 (0.71 to 0.86)	18% (13% to 22%)	Slope: 1.4 (0.9 to 1.9), p=0.11
Oostenbrink clinical	0.69 (0.57 to 0.81)	24% (19% to 29%)	Slope: 0.14 (0.09 to 0.2), p<0.001
Oostenbrink CSF	0.77 (0.66 to 0.87)	12% (8% to 17%)	Slope: 0.68 (0.43 to 0.93), p<0.001
Spanos LRM	0.74 (0.64 to 0.84)	20% (15% to 25%)	Slope: 0.4 (0.3 to 0.5), p<0.001

AUC, area under the curve; CSF, cerebrospinal fluid; LRM, logistic regression modelNA, not applicable

Median sensitivity of the 20 models was 80% (IQR 68%–91%) overall (table 5). NPV was ≥99% in 3/20 (15%) models overall.^30 35 46^ None of the models showed a sensitivity and NPV of 100% in all children.

**Table 5:** Sensitivity, specificity and predictive values for all children

	Sensitivity (95% CI)	Specificity (95% CI)	PPV (95% CI)	NPV (95% CI)
Bonsu	92% (89% to 94%)	20% (16% to 24%)	8% (5% to 11%)	97% (95% to 99%)
Bonsu 2	90% (89% to 93%)	60% (55% to 64%)	14% (11% to 17%)	99% (97% to 100%)
Boyer	87% (84% to 90%)	60% (56% to 65%)	14% (10% to 17%)	98% (97% to 100%)
Boum	83% (79% to 87%)	35% (30% to 39%)	8% (6% to 11%)	97% (95% to 98%)
Brivet	94% (92% to 97%)	11% (8% to 14%)	7% (5% to 9%)	96% (95% to 98%)
Chavanet—adults	71% (66% to 75%)	91% (89% to 94%)	37% (32% to 41%)	98% (96% to 99%)
Chavanet—children	77% (73% to 81%)	80% (76% to 84%)	22% (18 to 26%)	98% (96% to 99%)
De Cauwer	97% (95% to 98%)	50% (45% to 54%)	12% (9% to 15%)	100% (99% to 100%)
Deivanayagam	24% (20% to 28%)	100% (99% to 100%)	85% (81% to 88%)	95% (93 t%o 97%)
Hoen	76% (71% to 80%)	77% (74% to 81%)	19% (16% to 23%)	98% (96% to 99%)
Huang—model 1	80% (76% to 84%)	91% (88% to 94%)	39% (35% to 44%)	98% (97% to 100%)
Huang—model 2	83% (80% to 87%)	90% (87% to 93%)	37% (33% to 42%)	99% (99% to 100%)
Mirkhani—model 1	33% (29% to 38%)	97% (95% to 99%)	44% (40% to 49%)	95% (93% to 97%)
Mirkhani—model 2	30% (26% to 34%)	99% (98% to 100%)	61% (56% to 66%)	95% (93% to 97%)
Mwaniki	95% (93% to 97%)	14% (11% to 17%)	7% (5% to 10%)	97% (96 %to 99%)
Nigrovic	94% (91% to 96%)	51% (46% to 56%)	12% (9% to 15%)	99% (98% to 100%)
Oostenbrink combined*	74% (69% to 80%)	44% (40% to 50%)	9% (6% to 12%)	96% (94% to 98%)
Spanos CSF predictors	54% (49% to 59%)	92% (89% to 94%)	32% (27% to 36%)	97% (95% to 98%)
Tokuda	58% (52% to 64%)	73% (69% to 78%)	14% (10% to 18%)	96% (94% to 98%)
Wang	80% (76% to 83%)	60% (56% to 65%)	12% (9% to 16%)	98% (96% to 99%)

* Adjusted cut-off.

CSF, cerebrospinal fluid NPV, negative predictive value; PPV, positive predictive value

Median specificity was 67% (IQR 49%–91%) overall. Highest specificity was reached by the model of Deivanayagam (100%, 95% CI 99% to 100%) and the second model of Mirkhani (99%, 95% CI 98% to 100%) overall. Sensitivity of these models was only 24% (95% CI 20% to 28%) and 30% (95% CI 26% to 34%), respectively. Performance of models that were originally developed in children or neonates (n=13) differed from models developed in adults (n=3) or both children and adults (n=4), with median sensitivity in child models of 83% (IQR 80%–92%) compared with 58% (IQR 44%–74%) in (child and) adult models, and median specificity of 60% (IQR 44%–80%) in child models compared with 91% (IQR 75%–95%) in models developed in adults or both. The combination of sensitivity and specificity was best in the models of de Cauwer (resp. 97%, 50%) and Nigrovic (resp. 94%, 51%), and both showed an NPV of ≥99%.

#### Neonates

Discrimination was excellent in 2 models in neonates and good in 4 out of 13 models. The AUCs ranged from 0.58 to 0.91 (median 0.79, IQR 0.75–0.82, online supplemental table S6). Calibration in the large showed overestimation or underestimation in all of the models validated in the neonate cohort. Median sensitivity of the 20 models was 78% (IQR 59%–90%, online supplemental table S7). None of the models showed a sensitivity of 100%. Median specificity was 70% (IQR 45%–88%) in neonates. Only the model of Deivanayagam showed a specificity of 100% (95% CI 99% to 100%), with a sensitivity of 17% (95% CI 11% to 23%). Models developed in neonates (n=3, median sensitivity 88% (IQR 81%–88%), median specificity 86% (IQR 67%–88%)) overall performed better compared with models that were developed in children or adults or both (n=17, median sensitivity 73% (IQR 53%–91%), median specificity 52% (IQR 43%–88%)) in this cohort of neonates.

#### Children ≥28 days of age

Discrimination was excellent in six models and good in six models in children ≥28 days of age (online supplemental table S8). The AUCs ranged from 0.74 to 0.96 (median 0.89, IQR 0.82–0.92). Calibration in the large showed overestimation or underestimation in all of the models; however, the CSF model of Oostenbrink and the model of Boyer showed reasonable calibration with a slope of 1.1 (95% CI 0.6 to 1.5) and 1.0 (95% CI 0.5 to 1.4), respectively. Median sensitivity of the models was 82% (IQR 70%–93%). One model (De Cauwer) showed a 100% sensitivity, however with a specificity of 50% (IQR 44%–56%, online supplemental table S9). Moreover, nine models in this cohort showed a NPV of 99% or higher. Median specificity was 70% (IQR 48%–92%) in this cohort.

Models developed in children ≥28 days of age, adults or both (n=17, median sensitivity 85% (IQR 64–93), median specificity 65% (IQR 40–92)) overall showed higher sensitivity but lower specificity compared with models developed in neonates (n=3, median sensitivity 79% (IQR 75–84), median specificity 92% (IQR 74–92)) in this cohort of children aged ≥28 days of age.

## Discussion

We validated 23 clinical and laboratory-based diagnostic prediction models for bacterial meningitis, identified in a systematic review, using a cohort of 450 children with a suspected CNS infection. Quality of the included studies varied widely regarding study design, statistical analyses and reporting on model-building procedures. Discrimination was excellent in two models and good in 6 out of 13 models (4 out of 13 in neonates). Calibration showed relevant overestimation or underestimation of BM by all models. However, a sensitivity of 100% is required for implementation in clinical care due to the devastating consequences of missing this disease. Therefore, the results of these prediction models should always be incorporated with all information from the patient’s history, physical examination and ancillary investigations, and although some of these models provide valuable diagnostic information, they should not be used as a stand-alone test.

Children models performed worse in our children cohort compared with the adult cohort in which they were validated previously.^8^ Moreover, all models validated in this study performed worse than in their original publication. This is likely largely due to differences in case-mix between our validation cohort and the original derivation cohorts. Patient’s age differed significantly between the derivation cohorts, ranging from models derived in preterm neonates only to patients of all ages or adults, whereas our validation cohort consisted of children aged 0–18 years old. Even though discrimination overall was good to excellent, we show calibration was poor, which can be expected and can have many causes. Often, patient characteristics change over time and disease incidence or prevalence rates can vary between different populations, healthcare settings and countries. When an algorithm is developed in a cohort with a high incidence of BM, it may systematically give overestimated risk estimates (poor calibration in the large) when used in a setting with a lower BM incidence, as is the case in this study. Moreover, BM symptoms can vary greatly between different age groups. For example, neonates often show very non-specific signs and symptoms in meningitis, resulting in a different effect of clinical predictors and therefore poorer calibration slopes when validating a clinical characteristics-based models in this population. Other common causes of reduced discrimination and calibration are related to methodological problems regarding the algorithm itself, such as statistical overfitting. When using these models in new populations, it is important to take into account that the incidence of the outcome in the new population, as well as the different effect of predictors in this new population, will result in varying calibration, depending on the population in which the model is used. Before applying a model to a new population, recalibration of the model will sometimes be needed to adjust for these differences.

We chose to validate the prediction models in a broad cohort of patients with a suspected CNS infection because this is the population in which these models will be used in daily practice Therefore, validation in such a cohort provides a realistic view. Moreover, it shows that these models cannot be directly applied to all new populations without validating and adjusting the model to the specific characteristics of the population accordingly.

The question also remains if machine-learning-based algorithms outperform clinical judgement. A systematic review on comparing diagnostic prediction models with clinical judgement for various medical conditions found that prediction models reduced the proportion of missed diagnoses in only 2 out of 46 publications.^68^ This was offset by a larger amount of false positives as well. Comparing the combination of clinical judgement assisted by prediction rules to clinical judgement alone would provide the most valuable information on the added value of prediction models on patient outcome, but studies on this topic are lacking thus far.

To date, a large amount of prediction models for BM have been developed but none showed excellent discrimination and calibration when validated in a broader population of all patients suspected of a CNS infection.

However, comparing the discriminative performance of prediction models for BM to an ideal diagnostic test with excellent discrimination might not be fair, since a diagnostic test for paediatric BM with 100% sensitivity and 100% specificity does not exist in clinical practice. All current tests show limitations that should be taken into account when assessing the results of an individual patient. Diagnostic prediction models could aid in addition to other diagnostic investigations, however should not be used on their own.

Future research should also focus on different ways of improving diagnosis in paediatric BM. Better biomarker-based point-of-care tests that can accurately exclude and include BM in children are needed, especially in complex cases in which definite diagnosis is still unclear after conventional CSF examination.

Our research has some limitations. First, some models included variables that were not available in our dataset. These models were validated without those variables, which could lead to difference in performance. Second, a substantial proportion of data were missing and had to be imputed. Although 30 different imputation sets were used, this could have led to some distortion of the performance measurements. Third, the number of patients with BM in our validation cohort was limited. Our validation cohort included 450 patients, including 30 patients with BM (7%), possibly leading to less reliable and generalisable results. When including a small number of cases, the findings are more susceptible to random variation. Because CIs were broad, performance in larger cohorts could find better performance. Last, 18 of the total of 468 (4%) episodes could not be included due to lack of data on predictor or outcome variables. Lack of data in the paper files of these episodes could be a result of an acute setting with an ill patient resulting in less time for data documentation, possibly influencing the amount of severely ill (BM) patients.

In conclusion, this review analysed 40 articles on diagnostic prediction models for BM in children and validated 23 prediction models in a multicentre prospective cohort of 450 children suspected of a CNS infection. The models showed good to excellent diagnostic accuracy with poor calibration in all models. Diagnostic prediction models could be of help in the diagnostic workup of paediatric BM but are not recommended to use on their own in routine individual patient care. Future research should focus on the added value of prediction models in clinical practice.

## supplementary material

10.1136/bmjopen-2023-081172 online supplemental file 1

## Data Availability

Data are available upon reasonable request.
