# Supplementary material for: Diagnostic prediction models for bacterial meningitis in children with a suspected central nervous system infection: a systematic review and prospective validation study
Source: BMJ Open. 2024 Aug 7;14(8):e081172. doi: 10.1136/bmjopen-2023-081172 (PMC11404199; doi:10.1136/bmjopen-2023-081172)
Supplement: online supplemental file 1 [file bmjopen-14-8-s001.pdf]

SUPPLEMENTARY MATERIAL

Paper title: Diagnostic prediction models for bacterial meningitis in children with a suspected central nervous system infection: a systematic review and prospective validation study

Authors: N.S. Groeneveld[1,2], M.W. Bijlsma[2,3], I.E. van Zeggeren[1,2], S.L. Staal[1,2], M.W.T. Tanck[4], D. van de Beek[1,2], M.C. Brouwer[1,2]

[1]Amsterdam UMC, University of Amsterdam, Department of Neurology, Amsterdam Neuroscience, Meibergdreef, Amsterdam, The Netherlands; Amsterdam UMC, University of Amsterdam,

[2]Amsterdam UMC, University of Amsterdam, Netherlands Reference Laboratory for Bacterial Meningitis, Amsterdam, The Netherlands;

[3]Amsterdam UMC, University of Amsterdam, Department of Pediatrics, Amsterdam Neuroscience, Meibergdreef, Amsterdam, The Netherlands; Amsterdam UMC, University of Amsterdam;

[4]Amsterdam UMC, University of Amsterdam, Department of Epidemiology, and Bioinformatics and Data Science, Amsterdam Public Health (APH), Meibergdreef, Amsterdam

Contents

PRISMA checklist

TRIPOD-SRMA checklist

STROBE checklist

Supplementary Methods

Literature search

List of variables used for imputation

Supplementary Tables

Supplementary Table S1 - Quality of included studies according to TRIPOD criteria

Supplementary Table S2 – Percentage missing data per variable

Supplementary Table S3 - Proportion bacterial meningitis per risk category in all children

Supplementary Table S4 – Discrimination and calibration for neonates

Supplementary Table S5 - Sensitivity, specificity and predictive values for neonates

Supplementary Table S6 - Discrimination and calibration for children ≥ 28 days of age

Supplementary Table S7 - Sensitivity, specificity and predictive values for children ≥ 28 days of age

Supplementary Table S8 - Discrimination and calibration for all patients (CSF leukocytes not corrected for erythrocyte count)

Supplementary Table S9 - Sensitivity, specificity and predictive values for all patients (CSF leukocytes not corrected for erythrocyte count)

Supplementary Figures

Supplementary Figure S1 – Inclusion process

Supplementary Figure S2- Calibration curves of prediction model performance in all children

TRIPOD-SRMA checklist

Section and Topic	Item #	Checklist item	Location where item is reported
**TITLE**			
Title	1	Identify the report as a systematic review or meta-analysis (or both) of diagnostic or prognostic model studies. Specify the target population and outcome(s) predicted as relevant to the review question	1
**ABSTRACT**			
Abstract	2	See the TRIPOD-SRMA checklist for abstracts*.	3
**INTRODUCTION**			
Rationale	3	Describe the rationale for the review in the context of existing knowledge.	5
Objectives	4	Provide an explicit statement of the objective(s) being addressed with reference to: target population, index and comparator models (as relevant), outcome(s), time (prediction horizon and intended moment of using the model), and setting.	5
**METHODS**			
Study eligibility criteria	5	Specify study characteristics used as eligibility criteria, including any prediction models of specific interest, and whether development or validation studies (or both) were eligible.	6
Information sources	6	Specify all databases, registers, websites, organisations, reference lists and other sources searched or consulted to identify studies. Specify the date when each source was last searched or consulted.	6
Search strategy	7	Present the full search strategies for all databases, registers and websites, including any filters and limits used.	Supplementary methods
Study selection process	8	Specify the methods used to decide whether a study met the inclusion criteria of the review, including how many reviewers screened each record and each report retrieved, whether they worked independently, and if applicable, details of automation tools used in the process.	6
Data collection process	9	Specify the methods used to collect data from reports, including how many reviewers collected data from each report, whether they worked independently, any processes for obtaining or confirming data from study investigators, and if applicable, details of automation tools used in the process.	6
Data items	10a	List and define all outcomes for which data were sought.	7,8
	10b	State the model performance measures that were sought (eg, measures of calibration, discrimination, overall model fit, clinical utility).	7,8
	10c	Describe how any desired but unreported data items (items 10a, 10b) were handled (eg, contacted authors, calculated from other reported information).	8
Risk of bias and	11	Specify the methods used to assess risk of bias in the included studies and their applicability to the review question. This should be	Supplementary

Section and Topic	Item #	Checklist item	Location where item is reported
applicability assessment		done separately for each model development and validation. Include details of any tool(s) used, how many reviewers assessed each study and whether they worked independently.	results
Synthesis methods	12a	Describe any methods for synthesising estimates of performance measures for each model. If meta-analysis was carried out, describe the methods used, including any transformations of data before pooling, how any heterogeneity in model performance was quantified and handled, and software package(s) used.	8
	12b	Describe any methods used to explore possible causes of heterogeneity in model performance (eg, subgroup analysis, meta-regression), including whether or not they were planned.	NA
	12c	Describe any sensitivity analyses conducted to assess robustness of the synthesised results.	7
Certainty assessment	13	Describe any methods used to assess certainty (or confidence) in the body of evidence for a prediction model	8
**RESULTS**			
Study selection	14	Describe the results of the search and selection process, from the number of records identified in the search to the number of studies and models included in the review, ideally using a flow diagram..	9, Figure 1
Study and model characteristics	15	Present study characteristics and model details extracted (as per item 10a), and cite the study reports.	9,10, Table 1
Risk of bias and applicability	16	Present results of risk of bias and applicability assessment. This should be done separately for each model development and validation in each included study.	Table S1
Results of model performance in individual studies	17	Present performance estimates and confidence intervals for each model and all evaluations, including whether they relate to the internal or external validation performance. If internal, give details of the method.	Table 2
Results of syntheses	18a	Present the results of any synthesis of model performance, together with details of which study estimates contributed. If meta-analysis was carried out, then for each model and performance measure, present summary results, confidence/credible intervals, and measures of heterogeneity. Forest plots may be useful.	Table 4,5
	18b	For each model, present results of all investigations of possible causes of heterogeneity in model performance.	NA

Section and Topic	Item #	Checklist item	Location where item is reported
	18c	Present results of all sensitivity analyses conducted to assess the robustness of the synthesised results.	Table S3-S9
Certainty of evidence	19	Present assessments of certainty (or confidence) in the body of evidence for each prediction model of interest.	Table 4,5, S3-S9
**DISCUSSION**			
Summary of evidence, Limitations, Implications	20	Summarise the main findings including the strengths and limitations of the evidence	15-18
	21	Discuss the strengths and limitations of the review process.	NA
	22	Discuss implications of the results in the context of other evidence and for practice, policy, and future research.	16,17
**OTHER INFORMATION**			
Registration and protocol	23a	Provide registration information for the review, including register name and registration number, or state that the review was not registered.	NA
	23b	Indicate where the review protocol can be accessed, or state that a protocol was not prepared.	21
	23c	Describe and explain any amendments to information provided at registration or in the protocol.	NA
Support	24	Describe sources of financial or non-financial support for the review, and the role of the funders or sponsors in the review.	20,21
Competing interests	25	Declare any competing interests of review authors.	21
Availability of data, code and other materials	26	Report which of the following are publicly available and where they can be found: template data collection forms; data extracted from included studies; data used for all analyses; analytic code; any other materials used in the review.	21
NA = Not applicable, NR = not reported			

STROBE checklist

Our study is reported according to the Strengthening the Reporting of Observational Studies in Epidemiology (STROBE) statement. The STROBE checklist, downloaded from https://www.strobe-statement.org, is shown below.

	Item No	Recommendation	Manuscript page
**Title and abstract**	1	(*a*) Indicate the study's design with a commonly used term in the title or the abstract	1
		(*b*) Provide in the abstract an informative and balanced summary of what was done and what was found	3
**Introduction**			
Background/rationale	2	Explain the scientific background and rationale for the investigation being reported	5
Objectives	3	State specific objectives, including any prespecified hypotheses	5
**Methods**			
Study design	4	Present key elements of study design early in the paper	6
Setting	5	Describe the setting, locations, and relevant dates, including periods of recruitment, exposure, follow-up, and data collection	6,7
Participants	6	(*a*) Give the eligibility criteria, and the sources and methods of selection of participants. Describe methods of follow-up	6,7
		(*b*) For matched studies, give matching criteria and number of exposed and unexposed	NA
Variables	7	Clearly define all outcomes, exposures, predictors, potential confounders, and effect modifiers. Give diagnostic criteria, if applicable	6-8
Data sources/ measurement	8*	For each variable of interest, give sources of data and details of methods of assessment (measurement). Describe comparability of assessment methods if there is more than one group	6-8
Bias	9	Describe any efforts to address potential sources of bias	6
Study size	10	Explain how the study size was arrived at	NA
Quantitative variables	11	Explain how quantitative variables were handled in the analyses. If applicable, describe which groupings were chosen and why	6-8

Statistical methods	12		(*a*) Describe all statistical methods, including those used to control for confounding	7,8
			(*b*) Describe any methods used to examine subgroups and interactions	7,8
			(*c*) Explain how missing data were addressed	8,9
			(*d*) If applicable, explain how loss to follow-up was addressed	NA
			(*e*) Describe any sensitivity analyses	9
		**Results**		
Participants	13*		(a) Report numbers of individuals at each stage of study—eg numbers potentially eligible, examined for eligibility, confirmed eligible, included in the study, completing follow-up, and analysed	10,11
			(b) Give reasons for non-participation at each stage	10
			(c) Consider use of a flow diagram	NA
Descriptive data	14*		(a) Give characteristics of study participants (eg demographic, clinical, social) and information on exposures and potential confounders	10,11, Table 3
			(b) Indicate number of participants with missing data for each variable of interest	8,Table S2
			(c) Summarise follow-up time (eg, average and total amount)	NA
Outcome data	15*		Report numbers of outcome events or summary measures over time	11
Main results	16		(*a*) Give unadjusted estimates and, if applicable, confounder-adjusted estimates and their precision (eg, 95% confidence interval). Make clear which confounders were adjusted for and why they were included	11-15
			(*b*) Report category boundaries when continuous variables were categorized	11-15, Table 3
			(*c*) If relevant, consider translating estimates of relative risk into absolute risk for a meaningful time period	NA
Other analyses	17		Report other analyses done—eg analyses of subgroups and interactions, and sensitivity analyses	Supplementary Tables S3-S9
		**Discussion**		
Key results	18		Summarise key results with reference to study objectives	15

Limitations	19		Discuss limitations of the study, taking into account sources of potential bias or imprecision. Discuss both direction and magnitude of any potential bias	17, 18
Interpretation	20		Give a cautious overall interpretation of results considering objectives, limitations, multiplicity of analyses, results from similar studies, and other relevant evidence	15-18
Generalisability	21		Discuss the generalizability (external validity) of the study results	15-18
		**Other information**		
Funding	22		Give the source of funding and the role of the funders for the present study and, if applicable, for the original study on which the present article is based	20
		NA = not applicable		

Supplementary methods

Literature search

MEDLINE

((("Meningitis"[MeSH]) AND ((stratification OR "ROC Curve"[Mesh] OR discrimination OR discriminate OR c statistic OR c statistic OR area under the curve OR AUC OR calibration OR indices OR algorithm OR multivariable) OR (validate OR predict*[tiab] OR rule*) OR (predict* AND (outcome* OR risk* OR model*)) OR ((history OR variable* OR criteria OR scor* OR characteristic* OR finding* OR factor*) AND (predict* OR model* OR decision* OR identify OR prognosis)) OR (decision* AND (model* OR clinical* OR logistic models/)) OR (prognostic AND (history OR variable* OR criteria OR scor* OR characteristic* OR finding* OR factor* OR model*)))))

List of variables used for imputation

1. Age
2. Birth weight
3. Date admission
4. Final diagnosis
5. History - Otitis media
6. History -Sinusitis
7. History -Pneumonia
8. History -Endocarditis
9. History -HIV positive
10. History -Cancer
11. History -Diabetes mellitus
12. History -Prematurity
13. History -Splenectomy
14. History -Immunosuppressive therapy
15. Duration of symptoms
16. Headache
17. Fever
18. Rash
19. Purpura
20. Vomiting
21. Circulatory shock
22. Irritability
23. Temperature
24. Systolic blood pressure
25. Diastolic blood pressure
26. Heartrate
27. Bulging fontanel
28. Neck stiffness
29. GCS total score
30. Seizures
31. Focal neurological abnormalities
32. Aphasia
33. Ataxia
34. Cranial nerve palsy
35. Paresis arm
36. Paresis leg
37. Blood glucose
38. Blood leukocyte count
39. Blood granulocyte count
40. Blood C-reactive protein
41. Blood culture
42. CSF culture
43. CSF Gram stain
44. CSF granulocytes percentage
45. CSF granulocytes count
46. CSF lymphocytes count
47. CSF leukocytes count
48. CSF glucose
49. CSF total protein
50. CSF:plasma glucose ratio
51. CSF neutrophil-to-lymphocyte ratio

Supplementary tables

Table S1. Quality of included studies according to TRIPOD criteria

Section		Item	D/V	Checklist item	Abdelrahim	Boum	Chen	Cheng	Dalai	Delannoy	Huang	Li	Mintegi	Mirkhani	Mwanaki	Obiero	Pelkonen	Wang
**Title and abstract**	Title	1	D;V	Identify the study as developing and/or validating a multivariable prediction model, the target population, and the outcome to be predicted	p. 1	p.131	p. 1132	p. 1018	p. 99	p. 447	p.1	p.1	p. 1	p. 141	p. 1	p. 130	p. 462	p. 64
	Abstract	2	D;V	Provide a summary of objectives, study design, setting, participants, sample size, predictors, outcome, statistical analysis, results, and conclusions.	p. 1	p. 131	p. 1132	p. 1018	p. 99	p. 447	p. 1	p. 1	p. 1	p. 141	p. 1	p. 130	p. 462	p. 64
**Introduction**	Background and objectives	3a	D;V	Explain the medical context (including whether diagnostic or prognostic) and rationale for developing or validating the multivariable prediction model, including references to existing models	p. 2	p. 131	p. 1132, 1133	p. 1019	p. 100	p. 447	p. 2	p. 1,2	p. 2	p. 141, 142	p. 1,2	p. 130,131	p. 462	p. 64,65
		3b	D;V	Specify the objectives, including whether the study describes the development or validation of the model or both	p. 2	p. 131	p. 1133	p. 1019	p. 100	p. 448	p. 2	p. 1,2	p. 2	p. 142	p. 2	p. 131	p. 462	p. 65
**Methods**	Source of data	4a	D;V	Describe the study design or source data (e.g., randomized trial, cohort, or registry data), separately for the development and validation sets, if applicable	p. 2	p. 131	p. 1133	p. 1019	p. 100	p. 448	p. 2	p. 2	p. 2	p. 142	p. 2	p. 131	p. 462	p. 65
		4b	D;V	Specify the key study dates, including start of accrual, end of accrual and, if applicable, end of follow up	p. 2	p. 131	p. 1133	p. 1019	p. 100	p. 448	p. 2	p. 2	p. 2	p. 142	p. 2	p. 131	p. 462	p. 65
	Participants	5a	D;V	Specify key elements of the study setting (e.g., primary care, secondary care, general population) including number and location of centers	p. 2	p. 131	p. 1133	p. 1019	p. 100	p. 448	p. 2	p. 2	p. 2	x	p. 2	p. 131	p. 462	p. 65
		5b	D;V	Describe eligibility criteria for participants	p. 2	p. 131	p. 1133	p. 1019	p. 100	p. 448	p. 2	p. 2	p. 2	p. 142	p. 2	p. 131	p. 462	p. 65
		5c	D;V	Give details of treatments received, if relevant	NA	NA	NA	NA	NA	NA	NA	NA	NA	NA	NA	NA	NA	NA

	Outcome	6a	D; V	Clearly define the outcome that is predicted by the prediction model, including how and when assessed	p. 3	p. 131	1134	p. 1019	p. 100, 101	p. 448	p. 2	p. 2	p. 2	p. 142	p. 2	p. 131	p. 462	p. 66
		6b	D; V	Report any actions to blind assessment of predictors of the outcome to be predicted	NA	NA	NA	NA	NA	NA	NA	NA	NA	NA	NA	NA	NA	NA
	Predictors	7a	D; V	Clearly define all predictors used in developing or validating the multivariable prediction model, including how and when they were measured	p. 3	p. 131, 132	p. 1131	p. 1019, 1020	p. 100	p. 448	p. 2,3	p. 2	p. 3	p. 142	p. 2	p. 131	p. 462, 463	p. 64,65
		7b	D; V	Report any actions to blind assessment of predictors for the outcome and other predictors	NA	NA	NA	NA	NA	NA	NA	NA	NA	NA	NA	NA	NA	NA
	Sample size	8	D; V	Explain how the study size was arrived at	NR	NR	p. 1134	NR	p. 101	p. 449	NR	p. 3,4,5	p. 4	p. 142	NR	NR	NR	NR
		9	D; V	Describe how missing data were handled (e.g. complete-case analysis, single imputation, multiple imputation, with details of any imputation method)	NR	NR	p. 1134	NR	NR	NR	NR	p.3	NR	p. 142	NR	NR	NR	p. 67
	Statistical analysis methods	10a	D	Describe how predictors were handled in the analysis	NA	p. 131	p. 1134	p. 1020	p. 101	p. 449	p. 3	p. 3	p. 3	p. 142	p. 3	NA	p. 463	NA
		10b	D	Specify type of model, all model-building procedures (including any predictor selection), and method for internal validation	NA	p. 131	p. 1134	p. 1020	p. 101	p. 448, 449	p. 2,3	p. 3	p. 3,4	p. 142	p. 3	NA	p. 463	NA
		10c	V	For validation, describe how the predictions were calculated	p. 3	p. 131	p. 1134	NA	NA	p. 448, 449	NA	NA	p. 3	p. 143	p. 3	p. 131, 132	p. 462, 463	p.66
		10d	D; V	Specify all measures used to assess model performance and, if relevant, to compare with multiple models	NR	p. 132	p. 1136	p. 1020	p. 101	p. 448	p. 3	p.3	p. 3,4	p. 143	p. 3	p. 131	p. 463	p. 66
		10e	V	Describe any model updating (e.g., recalibration) arising from the validation, if done	NA	NA	NA	NA	NA	NA	NA	NA	NA	NA	NA	NA	NA	p. 66
	Risk groups	11	D; V	Provide details on how risk groups were created, if done	p. 3	p. 132	p. 1134	p. 1020	p. 101	p. 448, 449	p. 3	NA	p. 3,4, 5	p. 142, 143	p. 3	p. 131,132	NA	p. 66
	Development vs. Validation	12	V	For validation, identify any differences from the development data in setting, eligibility criteria, outcome and predictors	p. 1,2	p.131	p. 1140 (online)	NA	NA	p. 448	NA	NA	p. 2, 4	NR	p. 4,5	NR	NR	p. 66
**Results**	Participants	13a	D; V	Describe the flow of participants through the study, including number of participants with and without the	p. 3,4,5	p. 132	p. 1134, 1135	p. 1020	p. 101	p. 449, 450	p. 3	p. 3	p. 4	p. 142	p. 5	p. 132	Suppl. 2 (online)	p. 66

				outcome and, if applicable, a summary of the follow-up time														
		13b	D; V	Describe the characteristics of the participants (basic demographics, clinical features, available predictors), including the number of participants with missing data for predictors and outcome	p. 3,4,5,6	p. 132	p. 1135, 1140 (online)	p. 1020	p. 101, 102	p. 449	p. 2,3	p. 3	p. 4	p. 142	p. 3,4	p. 131, 132	Suppl. 2,3,4 (online)	p. 66, 67
		13c	V	For validation, show a comparison with the development data of the distribution of important variables (demographics, predictors and outcome)	NR	p. 131, 132	P. 1135, 1140 (online)	NA	NA	NR	NA	NA	p. 4	NR	p. 3,4,5,6	NR	NR	NR
	Model development	14a	D	Specify the number of participants and outcome events in each analysis	NA	p. 132	p. 1136	NA	NA	NA	p. 3	p. 8	NR	NR	p. 6,7	NA	NR	NA
		14b	D	If done, report the unadjusted association between each candidate predictor and outcome	NA	NA	p. 1136	NA	NA	NA	p. 4	p. 4	p. 5	NA	p. 6,7	NA	p. 463	NA
	Model specification	15a	D	Present the full prediction model to allow predictions for individuals (i.e., all regression coefficients, and model intercept or baseline survival at given time point)	NA	p. 132	p. 1134, 1135, 1136	p. 1022	p. 104	NA	p.5	p. 4	p. 5	p. 143	p. 8	NA	p. 473	NA
		15b	D	Explain how to use the prediction model	NA	p. 132	NR	p. 1020, 1021	NR	NA	p. 5	NR	p. 5	p. 143	p. 8	NA	NR	NA
	Model performance	16	D; V	Report performance measures (with CIs) for the prediction model	NR	p. 132	p. 1132, 1136	p. 1022, 1023	NR	p. 449,450	p. 5	p. 4	p. 5	p. 143	p. 8	p. 134	p. 463	p. 67,68
	Model-updating	17	V	If done, report the results from any model updating (i.e., model specification, model performance)	NA	p. 132	NA	NA	NA	NA	NA	NA	NA	NA	NA	NA	NA	p. 68
**Discussion**	Limitations	18	D; V	Discuss any limitations of the study (such as nonrepresentative sample, few events per predictor, missing data)	NR	p. 132	p. 1138, 1139	p. 1025	p. 104	p. 452	p. 6	p. 6	p. 6,7	p. 143	p. 9	p. 135	p. 465	p. 69
	Interpretation	19a	V	For validation, discuss the results with reference to performance in the development data, and any other validation data	p. 6,7,8	p. 132	NR	NA	NA	p. 451	NA	NA	p. 6,7	NA	p. 6	p. 134	p. 464	p. 68,69
		19b	D; V	Give an overall interpretation of the results, considering objectives, limitations, results from similar studies and other relevant evidence	p.6,7,8,9	p. 132	p. 1137, 1138, 1139	p. 1023, 1024, 1025	p. 103, 104	p. 451, 452	p. 4,5,6	p. 6	p. 6,7	p. 143	p. 6,7,8,9	p. 134, 135	p. 464, 465	p. 68,69
	Implications	20	D; V	Discuss the potential clinical use of the model and implications for future research	p.9	p. 132	p. 1138	p. 1023, 1024, 1025	p. 104	p. 451, 452	p. 5,6	p. 6	p. 5	p. 143	p.7,8,9	p. 134, 135	p. 465	p. 69,70

**Other information**	Supplementary information	21	D;V	Provide information about the availability of supplementary resources, such as study protocol, Web calculator and data sets	p.9	NA	p. 1140	NA	NA	NA	p. 6	p. 8,9	p. 4,5	NA	NA	p. 132-135	p. 462	NA
	Funding	22	D;V	Give the source of funding and the role of the funders for the present study	p.9	p. 133	p. 1132	p. 1025	NR	p. 452	NR	p.6	p. 8	NR	p. 9	p. 135	p. 462	p. 70
				**No. of reported (green) / no. of items applicable**	18/26 (70%)	25/31 (81%)	31/32 (97%)	23/25 (92%)	19/25 (76%)	25/27 (93%)	24/28 (96%)	25/27 (93%)	29/32 (91%)	19/29 (66%)	29/31 (94%)	22/26 (85%)	24/31 (77%)	25/27 (93%)

D= development, V= validation, CI= confidence interval. Green= reported, Orange= reported incomplete, Yellow/NA= not applicable, Red/NR= not reported

Table S2. Percentage missing data per variable

	% missing	Model using this variable
**Gender**	0	Li, Mentis, Spanos
**Months from August**	0	Spanos
**Age**	0.2	Bonsu, Freedman, Mentis, Pelkonen
**Birth weight**	87.8	Li, Pelkonen
**Duration of symptoms**	11.1	Oostenbrink, Pelkonen
**Fever**	7.1	Chen, Mwaniki
**Seizures**	12.4	Boyer, Brivet, Dubos, Mwaniki, Nigrovic, Pelkonen, Wang
**Vomiting**	12.4	Oostenbrink
**Irritability**	25.4	Chen, Dubos, Mwaniki
**Temperature**	20.4	Boyer, Mwaniki
**Purpura**	2.9	Boyer, Dubos, Oostenbrink
**Bulging fontanel**	89.2	Boum, Chen, Mwaniki
**Neck stiffness**	44.9	Boum, Oostenbrink
**Glasgow coma scale**	63.7	Brivet, Oostenbrink, Tokuda
**Cranial nerve palsy**	70.4	Boyer, Brivet
**Aphasia**	85.8	Boyer, Brivet
**Ataxia**	86.9	Boyer, Brivet
**Paresis**	11.8	Boyer, Brivet
**Blood CRP**	6.0	Chen, De Cauwer, Li, Mentis, Mintegi, Oostenbrink
**Blood glucose**	37.4	Hoen
**Blood leukocyte count**	4.6	Bonsu 2, Boyer, Chavanet, Hoen
**Blood granulocyte count**	16.8	Boum, Nigrovic, Wang
**CSF leukocyte count**	5.1	Bonsu 2, Boum, Boyer, Chavanet, Deivanayagam, Freedman, Huang, Li, Mentis, Mirkhani, Spanos
**CSF granulocyte count**	66.4	Bonsu, Bonsu 2, Boyer, Brivet, Chavanet, De Cauwer, Deivanayagam, Freedman, Hoen, Mentis, Mintegi, Nigrovic, Oostenbrink, Spanos, Tokuda, Wang
**CSF lymphocyte count**	95.6	Mentis
**CSF glucose**	5.8	Bonsu 2, Boyer, De Cauwer, Deivanayagam, Freedman, Huang, Li, Mirkhani, Spanos
**CSF: blood glucose ratio**	41.4	Chavanet, Deivanayagam, Freedman, Oostenbrink, Spanos
**CSF protein count**	6.9	Bonsu, Bonsu 2, Boyer, Chavanet, De Cauwer, Dubos, Freedman, Hoen, Huang, Mintegi, Mirkhani, Nigrovic, Spanos, Wang

CRP = C-reactive protein, CSF = cerebrospinal fluid

Table S3. Proportion bacterial meningitis per risk category in all children

**Prediction model**	**Proportion BM in our validation cohort (n=450) before using the prediction model is 7%.**	
	Percentage BM (95% CI) in low risk category	Percentage BM (95% CI) in high risk category
Bonsu	3.0 (1.4-4.6)	7.9 (5.3-11.0)
Bonsu 2	1.1 (0.2-2.1)	13.9 (10.7-17.1)
Boyer	1.5 (0.4-2.7)	13.6 (10.4-16.8)
Boum	3.4 (1.7-5.1)	8.3 (5.8-11.0)
Brivet	3.6 (1.9-5.4)	7.0 (4.7-9.4)
Chavanet - adults	5.2 (3.2-7.3)	55.3 (49.9.60.6)
Chavanet - children	2.0 (0.7-3.3)	21.8 (17.9-25.6)
De Cauwer	0.5 (-0.02-1.1)	12.1 (9.1-15.1)
Deivanayagam	5.2 (3.1-7.2)	84.8 (81.1-88.4)
Hoen	2.2 (0.8-3.6)	19.4 (15.8-23.1)
Huang – model 1	1.5 (0.4-2.7)	38.5 (34.0-43.0)
Huang – model 2	1.3 (0.3-2.4)	36.0 (32.0-40.4)
Mirkhani – model 1	4.7 (2.7-6.6)	44.3 (39.7-48.9)
Mirkhani – model 2	4.8 (2.8-6.8)	61.1 (56.4-66.0)
Mwanaki	2.7 (1.3-4.1)	7.3 (4.9-9.7)
Nigrovic	0.9 (0.03-1.8)	12.0 (9.0-15.0)
Oostenbrink combined	5.4 (3.3-7.5)	7.45 (5.0-9.9)
Spanos CSF predictors	3.5 (1.8-5.2)	32.0 (27.1-36.1)
Tokuda	4.1 (2.2-6.0)	14.3 (10.1-18.5)
Wang	2.4 (1.0-3.8)	12.5 (9.4-15.5)

BM denotes bacterial meningitis, CI = confidence interval, CSF = cerebrospinal fluid

Table S4. Discrimination and calibration for neonates

	AUC (95% CI)	Calibration in the large (95% CI)	Calibration slope (95% CI), p-value
**Bonsu**	0.78 (0.64-0.92)	28% (20 to 36%)	Slope: 0.08 (0.03 to 0.13), p<0.001
**Bonsu 2**	0.82 (0.70-0.93)	-8% (-12 to -3%)	Slope: 1.46 (0.79 to 2.15), p= 0.15
**Boyer**	0.80 (0.63-0.95)	2% (-5 to 8%)	Slope: 0.77 (0.30 to 1.23), p= 0.18
**Chavanet - adults**	0.85 (0.75-0.96)	NA	NA
**Chavanet - children**	0.79 (0.68-0.91)	NA	NA
**De Cauwer**	0.78 (0.68-0.88)	14% (7 to 22%)	Slope: 1.53 (0.72 to 2.34), p= 0.18
**Hoen**	0.80 (0.68-0.92)	7% (-0.2 to 14%)	Slope: 0.08 (0.01 to 0.16), p<0.001
**Huang - model 1**	0.91 (0.82-0.99)	NA	NA
**Huang - model 2**	0.91 (0.83-0.99)	NA	NA
**Nigrovic**	0.75 (0.65-0.85)	15% (7 to 22%)	Slope: 1.50 (0.65-2.35), p= 0.22
**Oostenbrink clinical**	0.58 (0.0.40-0.76)	13% (5 to 21%)	Slope: 0.06 (-0.1 to 0.20), p<0.001
**Oostenbrink CSF**	0.74 (0.58-0.90)	8% (1 to 16%)	Slope: 0.49 (0.12 to 0.85), p<0.05
**Spanos LRM**	0.69 (0.56-0.82)	11% (4 to 19%)	Slope: 0.30 (0.12 to 0.49), p<0.001

AUC = area under the curve, CI = confidence interval, NA = not applicable, CSF = cerebrospinal fluid, LRM = logistic regression model

Table S5. Sensitivity, specificity and predictive values for neonates

	Sensitivity (95% CI)	Specificity (95% CI)	PPV (95% CI)	NPV (95% CI)
**Bonsu**	97% (95-99%)	19% (13-25%)	11% (6-16%)	98% (97-100%)
**Bonsu 2**	89% (84-93%)	51% (44-59%)	16% (11-22%)	98% (96-100%)
**Boyer**	87% (82-93%)	52% (44-60%)	16% (11-22%)	98% (95-100%)
**Boum**	73% (65-81%)	41% (33-49%)	12% (7-17%)	93% (90-97%)
**Brivet**	91% (86-95%)	7% (3-10%)	9% (5-13%)	86% (79-92%)
**Chavanet - adults**	65% (58-72%)	88% (84-93%)	37% (30-45%)	96% (93-99%)
**Chavanet - children**	83% (77-89%)	72% (66-79%)	24% (18-31%)	98% (95-100%)
**De Cauwer**	94% (90-97%)	49% (41-56%)	16% (10-21%)	99% (97-100%)
**Deivanayagam**	17% (11-23%)	100% (99-100%)	94% (91-98%)	92% (88-96%)
**Hoen**	73% (66-79%)	79% (73-85%)	27% (20-34%)	97% (94-99%)
**Huang - model 1**	88% (83-93%)	88% (83-93%)	45% (37-52%)	99% (97-100%)
**Huang - model 2**	88% (83-92%)	86% (80-91%)	39% (32-47%)	99% (97-100%)
**Mirkhani - model 1**	46% (38-53%)	95% (92-98%)	51% (43-58%)	94% (91-98%)
**Mirkhani - model 2**	36% (29-44%)	98% (96-100%)	64% (57-72%)	94% (90-97%)
**Mwanaki**	93% (89-96%)	22% (16-29%)	11% (6-16%)	96% (94-99%)
**Nigrovic**	94% (91-98%)	43% (36-51%)	15% (9-20%)	99% (97-100%)
**Oostenbrink combined****	61% (58-77%)	45% (37-52%)	10% (6-15%)	92% (87-96%)
**Spanos CSF predictors**	47% (39-54%)	90% (86-95%)	34% (27-42%)	94% (91-98%)
**Tokuda**	53% (43-62%)	72% (64-79%)	17% (10-24%)	93% (90-97%)
**Wang**	73% (66-80%)	67% (60-74%)	19% (13-25%)	96% (93-99%)

CI = confidence interval, PPV = positive predictive value, NPV = negative predictive value, CSF = cerebrospinal fluid.

** Adjusted cut-off

Table S6. Discrimination and calibration for children ≥ 28 days of age

	AUC (95% CI)	Calibration in the large (95% CI)	Calibration slope (95% CI), p-value
**Bonsu**	0.74 (0.58-0.90)	38% (31 to 44%)	Slope: 0.05 (0.02 to 0.08), p < 0.001
**Bonsu 2**	0.91 (0.81-1.0)	-3% (-6 to -0.3%)	Slope: 1.6 (1.0 to 2.2), p= 0.04
**Boyer**	0.85 (0.72-0.98)	2% (-2 to 7%)	Slope: 1.0 (0.5 to 1.4), p= 0.76
**Chavanet - adults**	0.90 (0.79-1.0)	NA	NA
**Chavanet - children**	0.82 (0.68-0.96)	NA	NA
**De Cauwer**	0.92 (0.86-0.98)	18% (12 to 23%)	Slope: 2.6 (1.6 to 3.7), p < 0.001
**Hoen**	0.89 (0.81-0.97)	13% (8 to 19%)	Slope: 0.08 (0.04 to 0.12), p < 0.001
**Huang - model 1**	0.96 (0.93-0.99)	NA	NA
**Huang - model 2**	0.96 (0.94-0.99)	NA	NA
**Nigrovic**	0.82 (0.71-0.92)	20% (14 to 26%)	Slope: 1.4 (0.8 to 2.1), p= 0.20
**Oostenbrink clinical**	0.92 (0.85-0.98)	32% (26 to 38%)	Slope: 0.2 (0.2 to 0.3), p < 0.001
**Oostenbrink CSF**	0.87 (0.75-0.99)	15% (9 to 20%)	Slope: 1.1 (0.6 to 1.5), p = 0.73
**Spanos LRM**	0.80 (0.67-0.94)	25% (19 to 31%)	Slope: 0.5 (0.3 to 0.8), p < 0.001

AUC = area under the curve, CI = confidence interval, NA = not applicable, CSF = cerebrospinal fluid, LRM = logistic regression model

Table S7. Sensitivity, specificity and predictive values for children ≥ 28 days of age

	Sensitivity (95% CI)	Specificity (95% CI)	PPV (95% CI)	NPV (95% CI)
**Bonsu**	86% (81-90%)	22% (17-27%)	6% (3-9%)	96% (94-99%)
**Bonsu 2**	93% (89-96%)	65% (59-71%)	13% (9-16%)	99% (99-100%)
**Boyer**	87% (82-91%)	65% (59-71%)	11% (8-16%)	99% (98-100%)
**Boum**	96% (94-98%)	31% (25-36%)	7% (4-10%)	99% (99-100%)
**Brivet**	99% (98-99%)	13% (9-18%)	6% (3-9%)	99% (99-100%)
**Chavanet - adults**	79% (74-83%)	93% (90-96%)	38% (32-44%)	99% (97-100%)
**Chavanet - children**	71% (66-77%)	85% (80-89%)	20% (15-25%)	98% (97-100%)
**De Cauwer**	100% (99-100%)	50% (44-56%)	10% (6-13%)	100% (99-100%)
**Deivanayagam**	36% (30-41%)	100% (99-100%)	82% (77-87%)	97% (95-99%)
**Hoen**	79% (74-84%)	77% (72-82%)	15% (11-19%)	99% (97-100%)
**Huang - model 1**	71% (66-77%)	92% (89-96%)	34% (28-39%)	98% (97-100%)
**Huang - model 2**	79% (74-83%)	92% (88-95%)	34% (28-39%)	98% (97-100%)
**Mirkhani - model 1**	21% (17-26%)	98% (96-100%)	37% (30-42%)	96% (94-98%)
**Mirkhani - model 2**	21% (17-26%)	99% (98-100%)	58% (51-64%)	96% (94-98%)
**Mwanaki**	98% (96-99%)	9% (5-12%)	5% (3-8%)	98% (98-99%)
**Nigrovic**	93% (90-96%)	55% (49-61%)	10% (6-13%)	99% (98-100%)
**Oostenbrink combined***	86% (81-91%)	28% (22-33%)	6% (3-9%)	97% (96-99%)
**Spanos CSF predictors**	64% (59-70%)	92% (89-95%)	31% (26-37%)	98% (96-100%)
**Tokuda**	64% (57-71%)	74% (68-79%)	13% (8-18%)	97% (95-99%)
**Wang**	89% (85-93%)	56% (50-61%)	10% (6-13%)	99% (98-100%)

CI = confidence interval, PPV = positive predictive value, NPV = negative predictive value

* Adjusted cut-off

Table S8. Discrimination and calibration (CSF leukocytes not corrected for erythrocyte count)

	AUC (95% CI)	Calibration in the large (95% CI)	Calibration slope (95% CI), p-value
**Bonsu**	0.75 (0.65-0.86)	34% (29 to 39%)	Slope: 0.06 (0.03 to 0.09) , p<0.001
**Bonsu 2**	0.87 (0.79-0.94)	-5% (-8 to -2%)	Slope: 1.55 (1.11 to 1.99), p<0.001
**Boyer**	0.83 (0.72-0.93)	2% (-1 to 6%)	Slope: 0.86 (0.56 to 1.16), p= 0.34
**Chavanet - adults**	0.88 (0.81-0.96)	NA	NA
**Chavanet - children**	0.82 (0.73-0.90)	NA	NA
**De Cauwer**	0.85 (0.78-0.91)	16% (12 to 21%)	Slope: 2.0 (1.4 to 2.6), p<0.001
**Hoen**	0.84 (0.77-0.91)	11% (7 to 15%)	Slope: 0.07 (0.04 to 0.11), p < 0.001
**Huang - model 1**	0.93 (0.89-0.97)	NA	NA
**Huang - model 2**	0.93 (0.89-0.97)	NA	NA
**Nigrovic**	0.79 (0.72-0.86)	18% (13 to 23%)	Slope: 1.4 (0.9 to 1.9), p= 0.11
**Oostenbrink clinical**	0.70 (0.58-0.82)	25% (20 to 29%)	Slope: 0.15 (0.09 to 0.20), p<0.001
**Oostenbrink CSF**	0.81 (0.71-0.91)	12% (8 to 17%)	Slope: 0.76 (0.50 to 1.00), p= 0.057
**Spanos LRM**	0.74 (0.64-0.83)	19% (15 to 24%)	Slope: 0.40 (0.27 to 0.52), p<0.001

AUC = area under the curve, CI = confidence interval, NA = not applicable,

CSF = cerebrospinal fluid, LRM = logistic regression model

Table S9. Sensitivity, specificity and predictive values (CSF leukocytes not corrected for erythrocyte count)

	Sensitivity (95% CI)	Specificity (95% CI)	PPV (95% CI)	NPV (95% CI)
**Bonsu**	92% (89-94%)	20% (16-24%)	8% (5-11%)	97% (95-99%)
**Bonsu 2**	90% (89-93%)	60% (55-64%)	14% (11-17%)	99% (98-100%)
**Boyer**	87% (83-90%)	60% (56-65%)	14% (10-17%)	98% (97-100%)
**Boum**	85% (81-89%)	34% (30-39%)	8% (6-11%)	97% (95-99%)
**Brivet**	94% (92-97%)	11% (8-14%)	7% (5-9%)	96% (95-98%)
**Chavanet - adults**	71% (66-75%)	91% (89-94%)	37% (32-41%)	98% (96-99%)
**Chavanet - children**	77% (73-81%)	80% (76-84%)	22% (18-25%)	98% (97-99%)
**De Cauwer**	97% (95-98%)	50% (45-54%)	12% (9-15%)	100% (99-100%)
**Deivanayagam**	24% (20-28%)	99% (98-100%)	76% (72-80%)	95% (93-97%)
**Hoen**	76% (71-80%)	78% (74-81%)	19% (16-23%)	98% (96-99%)
**Huang - model 1**	80% (76-84%)	89% (86-92%)	34% (30-39%)	98% (97-100%)
**Huang - model 2**	83% (80-87%)	87% (84-90%)	31% (27-35%)	99% (98-100%)
**Mirkhani - model 1**	33% (29-38%)	96% (95-98%)	40% (36-45%)	95% (93-97%)
**Mirkhani - model 2**	30% (26-34%)	98% (96-99%)	50% (44-54%)	95% (93-97%)
**Mwanaki**	95% (93-97%)	14% (11-17%)	7% (5-10%)	97% (96-99%)
**Nigrovic**	94% (91-96%)	51% (46-55%)	12% (9-15%)	99% (98-100%)
**Oostenbrink combined***	68% (63-73%)	41% (36-45%)	7% (5-10%)	95% (92-97%)
**Spanos CSF predictors**	54% (49-59%)	92% (89-94%)	32% (27-36%)	97% (95-98%)
**Tokuda**	60% (54-65%)	73% (68-77%)	15% (10-19%)	96% (94-98%)
**Wang**	80% (76-84%)	60% (55-65%)	13% (9-16%)	98% (96-99%)

CI = confidence interval, PPV = positive predictive value, NPV = negative predictive value, CSF = cerebrospinal fluid.

* Adjusted cut-off

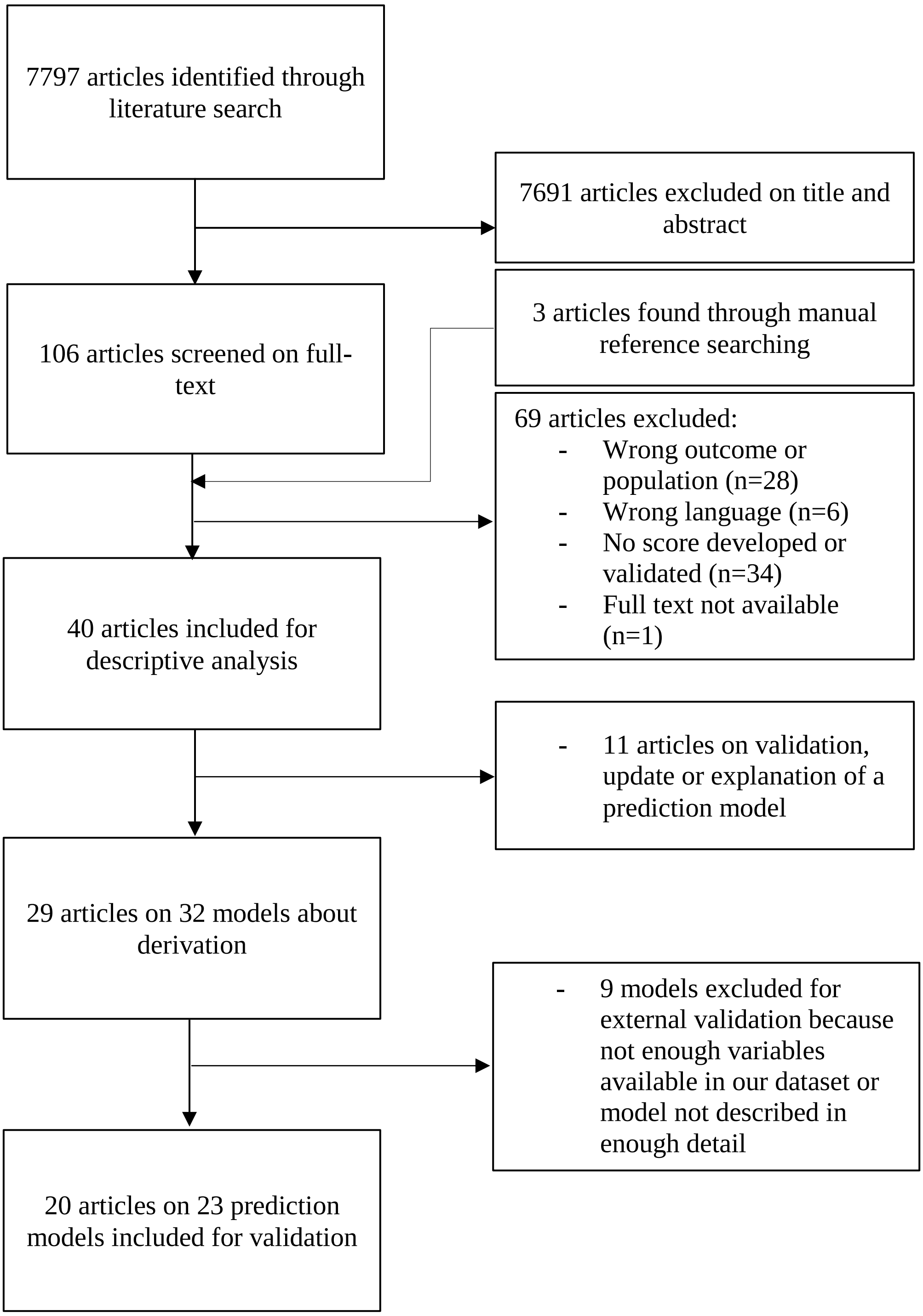

Figure S1. Inclusion process

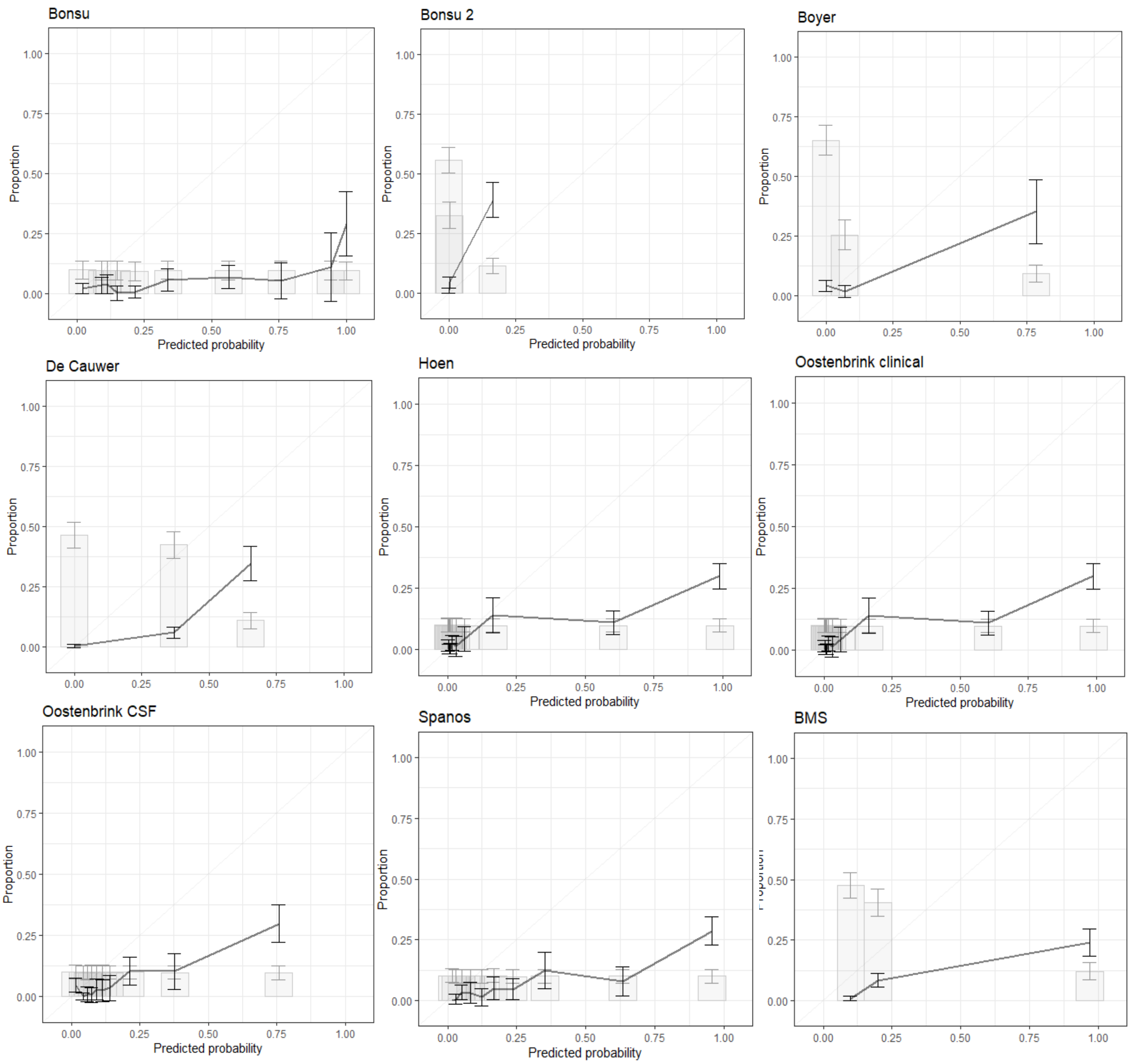

Figure S2. Calibration curves of prediction model performance in all children
