# Supplementary material for: Diagnostic prediction models for bacterial meningitis in children with a suspected central nervous system infection: a systematic review and prospective validation study
Source: BMJ Open. 2024 Aug 7;14(8):e081172. doi: 10.1136/bmjopen-2023-081172 (PMC11404199; doi:10.1136/bmjopen-2023-081172)
Supplement: Supplementary file 2 [file bmjopen-2023-081172.reviewer_comments.pdf]

PEER REVIEW HISTORY

BMJ Open publishes all reviews undertaken for accepted manuscripts. Reviewers are asked to complete a checklist review form (**http://bmjopen.bmj.com/site/about/resources/checklist.pdf**) and are provided with free text boxes to elaborate on their assessment. These free text comments are reproduced below.

ARTICLE DETAILS

**TITLE (PROVISIONAL)**	Diagnostic prediction models for bacterial meningitis in children with a suspected central nervous system infection: a systematic review and prospective validation study
**AUTHORS**	Groeneveld, Nina; Bijlsma, Merijn; van Zeggeren, Ingeborg; Staal, Steven; Tanck, Michael; van de Beek, Diederik; Brouwer, Matthijs

VERSION 1 – REVIEW

**REVIEWER**	Sergeant, Jamie<br>University of Manchester, Arthritis Research UK Centre for Epidemiology
**REVIEW RETURNED**	29-Nov-2023

**GENERAL COMMENTS**	This study is a systematic review of diagnostic clinical prediction models (CPMs) for bacterial meningitis (BM) in children with suspected meningitis, and also an external validation of these models in children with suspected central nervous system (CNS) infection. Attempting to report both a CPM systematic review and the external validation of multiple CPMs in a single manuscript is ambitious and I think that this has been achieved at the expense of detail. A meticulously conducted and reported systematic review together with a meticulously conducted and reported external validation study together would represent a major contribution in this area. I think each deserves their own manuscript, with sufficient detail to be reproducible, to be critically appraised and to be able to inform future research and practice.<br><br>Abstract: the acronym CNS is not defined in the abstract<br><br>p. 5 “Validation of prediction models in a broader population of patients suspected of a central nervous system (CNS) infection is necessary but is often lacking.”: Is “suspected meningitis” the same as “suspected CNS infection”? Please clarify.<br><br>p. 6 Methods: Systematic review: While it is welcome to see the PRISMA 2020 guidelines cited, there are now specific guidelines for the “Transparent reporting of multivariable prediction models for individual prognosis or diagnosis: checklist for systematic reviews and meta-analyses” (TRIPOD-SRMA, https://doi.org/10.1136/bmj-2022-073538). Adhering to these would improve the quality of the reporting.<br><br>p. 6 Methods: Systematic review: There is a surprising lack of detail on the methods of the systematic review, especially given that the PRISMA 2020 guidelines have been cited. Even fundamentals like the full details of the search terms and strategy are missing. I can see that some things are in the supplementary

material but they very likely belong in the main manuscript and are not even referenced there. These fundamentals are expected in any systematic review and are listed in both the PRISMA 2020 and TRIPOD-SRMA guidelines.

p. 6 “Article screening and data extraction were performed by one researcher (N.S.G.) and discrepancies were discussed and resolved by a second and third researcher (M.C.B and M.W.B)”: How can there be discrepancies if only one researcher screened and extracted?

p.6 “Quality of the included studies was assessed according to the Transparent Reporting of a multivariable prediction model for Individual Prognosis Or Diagnosis (TRIPOD) criteria”: I think you need to be clearer that you have assessed the quality of the reporting, not the quality of the studies. I recommend that you do assess the quality of the studies, in terms of their risk of bias and applicability, using the PROBAST (Prediction model Risk Of Bias ASsessment Tool). You can see a high-profile example of the use of PROBAST in the BMJ living systematic review “Prediction models for diagnosis and prognosis of covid-19: systematic review and critical appraisal” (https://doi.org/10.1136/bmj.m1328).

p. 6 “This was a multicentre prospective study in three hospitals”: Which hospitals? And when? I know you say that a detailed description of the cohort has been published previously, but the outline details are needed here. You should be adhering to the TRIPOD guidance for reporting prediction model validation (see https://www.equator-network.org/reporting-guidelines/tripod-statement/).

p. 7 “Patients with insufficient amount of data were excluded for this study.”: Please clarity the reasons for exclusion. For example, is this due to missing outcome data, predictor data, or something else?

p. 7 “The differences in baseline characteristics between bacterial meningitis and non-bacterial meningitis patients were identified with parametric and nonparametric tests.”: Why is this relevant if the aim is to externally validate existing models?

p. 8 “In prediction models based on a multivariable logistic regression model in which beta coefficients could not be retrieved from the original publication, we used the observed proportions for the different risk categories from the derivation study as expected proportions in the validation data.”: I’m confused by this. If a model is insufficiently specified (i.e. its full form including all coefficients and intercepts is not reported) then it is not possible to use this model to produce individual predictions. And without those individual predictions, how can you measure the performance of the model? The observed proportions are insufficient, and this does not constitute external validation. Did you attempt to contact the researchers who developed the inadequately reported models, in order to obtain their full functional form? If this is unsuccessful, you may have to reluctantly exclude these models from the external validation.

p. 8 “A probability of <0.1 was defined as low risk and >0.8 as high risk, based on agreement between two clinicians, in advance of the analysis. Probabilities of 0.1-0.8 were considered not significant for

	clinical decision making.": I'm not familiar with this clinical area but these risk thresholds really surprised me. Is it really true that a probability of 75% (or 50%, or 20%...) that a child has BM is of no consequence clinically? Maybe there's something context-specific that I don't understand.<br><br>p. 8 "Missing data were handled by multiple imputation using the R package MICE. We used 60 variables from medical history, physical examination and laboratory results as predictors to impute missing values": Further details of the imputation analyses should be provided, e.g. what imputation model was used? Also, did any of the development studies for the models address the issue of how to handle missing predictor data at application?<br><br>p. 8 "If a specific predictor from the model or a valid proxy, was not available in the PACEM dataset, the prediction model was validated without that particular variable": It's unclear how you can use a model to generate predictions when you do not have data on at least one of its predictor variables.<br><br>p. 9 The PRISMA flow diagram of study inclusion and exclusion is an important part of any systematic review and should not be relegated to the supplementary material.<br><br>p. 9 "A total of 23 models were developed in children…": 23 developed in children, 4 in both adults and children, and 3 in adults. That makes 30. Is there one missing?

**REVIEWER**	Shen, Susanne Dyckhoff<br>LMU Klinikum
**REVIEW RETURNED**	24-Jan-2024

**GENERAL COMMENTS**	The authors conducted a prospective validation study to assess diagnostic prediction models for bacterial meningitis, previously identified through a systematic literature review, in a cohort of 450 children with suspected CNS infection. While 2 models demonstrated an AUC over 0.90, none of the models achieved both a sensitivity of 100% and sufficiently high specificity. Moreover, all models performed less effectively compared to their original publication.<br><br>By comparing several diagnostic prediction models for bacterial meningitis in children, this valuable study holds significant clinical relevance. The prospective design of the validation cohort is of particular advantage. The authors raised interesting results and thoroughly discussed their implications.<br><br>Regarding the definition of bacterial meningitis (p. 8, lines 24ff) in the validation cohort, a few questions remain:<br>The authors defined bacterial meningitis in their validation cohort by 1) positive CSF culture, 2) positive blood culture with elevated CSF cell count or 3) negative CSF or blood culture, but elevated CSF leukocyte count and elevated infection parameters and clinical parameters associated with bacterial infection. Could authors please clarify for group 3 which blood infection parameters and their respective cut-offs as well as clinical signs were used to confirm bacterial meningitis? Were abnormal values for CSF protein and CSF glucose considered to define group 3? How were cases in this group distinguished from viral meningitis or encephalitis?

	In the enumeration of detected pathogens, it is noticeable that Streptococcus agalactiae was detected three times in CSF culture and four times in blood cultures. In the single case where a patient had Streptococcus agalactiae solely in the blood culture, without a corresponding detection in the CSF culture, this particular patient should also be included in the list of individual cases further below for the sake of completeness.<br><br>Furthermore, if the case numbers are sufficient, a subgroup analysis focusing on patients with confirmed pathogens for bacterial meningitis (groups 1 and 2, n=12 out of 30 patients) would be of great interest and could provide valuable insights.

**REVIEWER**	Oostenbrink, Rianne<br>Erasmus MC - Sophia Children's Hospital
**REVIEW RETURNED**	06-Feb-2024

**GENERAL COMMENTS**	The authors perform a nice study, to validate a large set of CPR on meningitis in a new cohort of patients. Design is appropriate. My main comment, is that salthough there are no real flaws in the analyses, the message of the paper from the analysis could be much clearer with a change in assessments. It is confusing that the authors mention CNS infections and BM, I assume they only use the BM as outcome for the validation, but it should be confirmed. Next, they report substantial problems in validation, but conclude that the models show good to excellent test results, so this seems contrasting. I would suggest that the authors assess better and discuss better their observations in the validation and the calibration results from the perspective of models being overfitted, differences in casesmix, and need for recalibration or remodelling. this now is mixed throughout the paper, and reads confusing. authors may consider to come with a message with more solutions<br><br>Specific comments:<br>abstract:<br>conclusion: impact on patient outcome is a different step in validation of CPRs (See Reilly et al, annals int med 2006), and is not the scope of this manuscript (no studies on impact analyses were included in the review)<br><br>Methods<br>p 7 line 5-6 'patients with insufficeint amount of data': how many were excluded, and please elaborate on impact of excluding these patients on the result<br>p7 line 8-9: sensitivity analysis should be in analysis/statistic section<br>p7 line 15-20: what is done with the cases of CNS infection, why mentioning this diagnosis separate?<br>p8 line 8 'diagnosis of bacterial meningitis based on positive CSF'....: this contrast with your presented definition of BM at page 7, so what definition did you use?<br>p8 line 40-41: prob< 0.1 low risk, > 0.8 high risk: the predicted risks of a CPR usually do not translate to actual risks perceived by the clinicians on an outcome. It would be more logic to use the threshold as set by the developers of the CPR for decisions in this assessment, rather than an overall prob of < 0.1 an d>0.8. otherwise, show that the thresholds set by the original authors cohere with these probabilities

	p8 line 57-59: excluding a variable from the CPR results by a reduced predicted risk per definition. so this should be corrected if you use subsequently the thresholds of the original authors, and will lead to need of calibration in the large. you could consider to adjust the pred risk by setting a mean value for the missing variable as observed in tht original cohort, thereby correcting the intercept<br><br>Results<br>p9 line 47-50: to assess the power of the developing study cohorts, please report the number of BM cases additional to the cohort size<br>p 10 line 47-48: please include definitions of the mentioned diagnoses in the paper/methods<br><br>p10 line 54: validation cohort includes 30 BM cases. usually it is adviced to include 100 cases and 100 noncases as a minimum for valid validation results. I understand the limitations of having such a cohort with BM. but at least, discuss the impact of this low number of cases on the power of your study, and how the results should be interpreted.<br>this in partciular applies to the sensitivity analyses, where the number of cases are much smaller!<br>p13 please report the number of BM cases in the two cohorts neonates and >28d given the remark above<br>discussion<br>p14 line 53-60 reduced performance is mentioned, but the cohort they are validated in is very different from the original cohorts for some (other ages, other inclusion criteria/severity level). it is logic that this provides a different mean risk, thereby in need for calibration in the large. this does not say any about the performance of the rules, but about the differences in the cohorts<br>p 15 lines 2-30: this deals with different origin of the populations. so please use in this discussion the results you observed from recalibration. you may add the assessments like Brier to discuss whether indeed differences are related to differences in case-mix versus overestimation in the original cohort. next, please take your small number of cases into account<br>p15 line 32 - 60: the topic machine learning seems a bit out of scope in this paper if there is only one machine learning model in the review?<br>p 16 line 45 : lead to difference in performance: see remarks before, this is a clear example that it is needed to explain that this could be corected by calibration in the large.<br>p 16 line 55: low number of cases is a real limitation of the paper, and should be discussed better<br><br>conclusion<br>p17 line 20-21 future resaerch focussin on the added value / impact (?) of CPRS: by this impact analyses is meant, this is not a result from current study, but could result from a systematic review identifying how many CPRs underwent impact analyses

VERSION 1 – AUTHOR RESPONSE

Reviewer: 1
Dr. Jamie Sergeant, University of Manchester, Manchester Academic Health Science Centre
Comments to the Author:

This study is a systematic review of diagnostic clinical prediction models (CPMs) for bacterial meningitis (BM) in children with suspected meningitis, and also an external validation of these models in children with suspected central nervous system (CNS) infection. Attempting to report both a CPM systematic review and the external validation of multiple CPMs in a single manuscript is ambitious and I think that this has been achieved at the expense of detail. A meticulously conducted and reported systematic review together with a meticulously conducted and reported external validation study together would represent a major contribution in this area. I think each deserves their own manuscript, with sufficient detail to be reproducible, to be critically appraised and to be able to inform future research and practice.

- Thank you, we agree two manuscripts would allow a more in-depth discussion but think that this single manuscript does justice to our main findings.

Abstract: the acronym CNS is not defined in the abstract

- Thank you for pointing this out. We have added this to the abstract.

p. 5 "Validation of prediction models in a broader population of patients suspected of a central nervous system (CNS) infection is necessary but is often lacking.": Is "suspected meningitis" the same as "suspected CNS infection"? Please clarify.

- Patients with a suspected CNS infection cover a more heterogeneous group than suspected meningitis alone, including bacterial meningitis, viral meningitis but also viral encephalitis. A clinical distinction between suspected meningitis and CNS infections in general is difficult if not impossible. Therefore we chose a broad population of all patients suspected of a CNS infection, which is likely to be the population in which the models will be used. Validation in this population consequently prevents overestimation of the performance of the model.

p. 6 Methods: Systematic review: While it is welcome to see the PRISMA 2020 guidelines cited, there are now specific guidelines for the "Transparent reporting of multivariable prediction models for individual prognosis or diagnosis: checklist for systematic reviews and meta-analyses" (TRIPOD-SRMA, https://doi.org/10.1136/bmj-2022-073538). Adhering to these would improve the quality of the reporting.

- We have replaced the PRISMA checklist with the TRIPOD-SRMA checklist in the supplementary material as suggested, and have adjusted the manuscript and abstract according to the TRIPOD-SRMA guidelines.

p. 6 Methods: Systematic review: There is a surprising lack of detail on the methods of the systematic review, especially given that the PRISMA 2020 guidelines have been cited. Even fundamentals like the full details of the search terms and strategy are missing. I can see that some things are in the supplementary material but they very likely belong in the main manuscript and are not even referenced there. These fundamentals are expected in any systematic review and are listed in both the PRISMA 2020 and TRIPOD-SRMA guidelines.

- We describe our detailed search terms in the supplementary material (page 10) and have added the detailed search from the previously validated search filter for prediction models as suggested.(1)

p. 6 “Article screening and data extraction were performed by one researcher (N.S.G.) and discrepancies were discussed and resolved by a second and third researcher (M.C.B and M.W.B)”: How can there be discrepancies if only one researcher screened and extracted?

- We agree the formulation was unclear and incorrect. We changed this phrase to: “Article screening and data extraction were performed by one researcher (N.S.G.) and when in doubt, NSG discussed this with a second and third researcher (M.C.B and M.W.B)”.

p.6 “Quality of the included studies was assessed according to the Transparent Reporting of a multivariable prediction model for Individual Prognosis Or Diagnosis (TRIPOD) criteria”: I think you need to be clearer that you have assessed the quality of the reporting, not the quality of the studies. I recommend that you do assess the quality of the studies, in terms of their risk of bias and applicability, using the PROBAST (Prediction model Risk Of Bias ASsessment Tool). You can see a high-profile example of the use of PROBAST in the BMJ living systematic review “Prediction models for diagnosis and prognosis of covid-19: systematic review and critical appraisal” (https://doi.org/10.1136/bmj.m1328).

- We have changed this sentence into: “Quality **of the reporting** of the included studies was assessed..”

p. 6 “This was a multicenter prospective study in three hospitals”: Which hospitals? And when? I know you say that a detailed description of the cohort has been published previously, but the outline details are needed here. You should be adhering to the TRIPOD guidance for reporting prediction model validation (see https://www.equator-network.org/reporting-guidelines/tripod-statement/).

- We have added details on the hospitals and inclusion dates to the paragraph ‘validation cohort’.

p. 7 “Patients with insufficient amount of data were excluded for this study.”: Please clarity the reasons for exclusion. For example, is this due to missing outcome data, predictor data, or something else?

- A more detailed description of patients that were excluded with reasons for exclusion was presented in the results (description of cohort paragraph, second sentence).

p. 7 “The differences in baseline characteristics between bacterial meningitis and non-bacterial meningitis patients were identified with parametric and nonparametric tests.”: Why is this relevant if the aim is to externally validate existing models?

- We agree that this is not relevant as an external validation of the existing models. We present baseline characteristics of our cohort and describe the differences between the bacterial meningitis and non-bacterial meningitis patients, including statistical testing of these differences in our cohort.

p. 8 “In prediction models based on a multivariable logistic regression model in which beta coefficients could not be retrieved from the original publication, we used the observed proportions for the different risk categories from the derivation study as expected proportions in the validation data.”: I’m confused by this. If a model is insufficiently specified (i.e. its full form including all coefficients and intercepts is not reported) then it is not possible to use this model to produce individual predictions. And without those individual predictions, how can you measure the performance of the model? The observed

proportions are insufficient, and this does not constitute external validation. Did you attempt to contact the researchers who developed the inadequately reported models, in order to obtain their full functional form? If this is unsuccessful, you may have to reluctantly exclude these models from the external validation.

- We agree that validation of a model that has not been fully specified is suboptimal. We decided upon this strategy based on previous work and have used this strategy in two models.(2) We have added an explanation about this to the methods section.

As example, see the article of Bonsu (2008), table 3. In this table we can see the number of bacterial meningitis cases per risk category (compared to the non-cases). We then calculated this proportions (0/333 for prediction score 0, 1/332 for prediction score 1, 3/127 for score 2, etc) and used these to calculate predicted probability.

- We did indeed contact authors of prediction models when information was not clear or missing, and added this to our methods section.

p. 8 "A probability of <0.1 was defined as low risk and >0.8 as high risk, based on agreement between two clinicians, in advance of the analysis. Probabilities of 0.1-0.8 were considered not significant for clinical decision making.": I'm not familiar with this clinical area but these risk thresholds really surprised me. Is it really true that a probability of 75% (or 50%, or 20%...) that a child has BM is of no consequence clinically? Maybe there's something context-specific that I don't understand.

- What we attempted to show is how often and by how much the risk of bacterial meningitis changed from the a-prior risk, depending on the outcome of the risk score. However, we agree with the reviewer these cut-off values are arbitrary and that pre-defined levels of clinical relevance cannot be stated. We have deleted these analyses from our manuscript.

To give an impression of the change from baseline risk of bacterial meningitis depending on the result (high risk or low risk) from the risk score, we have added supplementary table S3.

p. 8 "Missing data were handled by multiple imputation using the R package MICE. We used 60 variables from medical history, physical examination and laboratory results as predictors to impute missing values": Further details of the imputation analyses should be provided, e.g. what imputation model was used? Also, did any of the development studies for the models address the issue of how to handle missing predictor data at application?

- We have reassessed this list and found that we actually have used 51 variables for imputation. We have added this list of variables that were used to predict the value of the missing values to the supplementary data and corrected this in the manuscript. None of the articles describing the development of a prediction model report on how to handle missing variables when used in clinical practice.

p. 8 "If a specific predictor from the model or a valid proxy, was not available in the PACEM dataset, the prediction model was validated without that particular variable": It's unclear how you can use a model to generate predictions when you do not have data on at least one of its predictor variables.

- If a single predictor was missing in our dataset and the other x-y predictors were available, we decided to exclude that variable from the model. Although we agree that this is suboptimal, we think that validation of the limited model is more informative than no validation at all. We have also described this in our discussion of the limitations of our study.

p. 9 The PRISMA flow diagram of study inclusion and exclusion is an important part of any systematic review and should not be relegated to the supplementary material.

- We have now included the PRISMA flow diagram in the main manuscript (Figure 1) and have changed the numbers on included prediction models for validation due to changed made after comments of other reviewers.

p. 9 “A total of 23 models were developed in children…”: 23 developed in children, 4 in both adults and children, and 3 in adults. That makes 30. Is there one missing?

- Thank you for pointing out this typo, it should indeed be “Five models were developed in both adults and children”, instead of four. We corrected this and now it adds up to 30.

Reviewer: 2
Dr. Susanne Dyckhoff Shen, LMU Klinikum
Comments to the Author:
The authors conducted a prospective validation study to assess diagnostic prediction models for bacterial meningitis, previously identified through a systematic literature review, in a cohort of 450 children with suspected CNS infection. While 2 models demonstrated an AUC over 0.90, none of the models achieved both a sensitivity of 100% and sufficiently high specificity. Moreover, all models performed less effectively compared to their original publication.

By comparing several diagnostic prediction models for bacterial meningitis in children, this valuable study holds significant clinical relevance. The prospective design of the validation cohort is of particular advantage. The authors raised interesting results and thoroughly discussed their implications.

- Thank you

Regarding the definition of bacterial meningitis (p. 8, lines 24ff) in the validation cohort, a few questions remain:
The authors defined bacterial meningitis in their validation cohort by 1) positive CSF culture, 2) positive blood culture with elevated CSF cell count or 3) negative CSF or blood culture, but elevated CSF leukocyte count and elevated infection parameters and clinical parameters associated with bacterial infection. Could authors please clarify for group 3 which blood infection parameters and their respective cut-offs as well as clinical signs were used to confirm bacterial meningitis? Were abnormal values for CSF protein and CSF glucose considered to define group 3? How were cases in this group distinguished from viral meningitis or encephalitis?

- We have added the blood parameters that were taken into account, including the cut-offs. The diagnosis of this third group was also influenced by the final diagnosis of the treating physician, and we have added this information to the paragraph as well.

In the enumeration of detected pathogens, it is noticeable that Streptococcus agalactiae was detected three times in CSF culture and four times in blood cultures. In the single case where a patient had Streptococcus agalactiae solely in the blood culture, without a corresponding detection in the CSF

culture, this particular patient should also be included in the list of individual cases further below for the sake of completeness.

- In this specific patient the CSF culture failed due to a traumatic lumbar puncture, we have added this information to the list of individual cases.

Furthermore, if the case numbers are sufficient, a subgroup analysis focusing on patients with confirmed pathogens for bacterial meningitis (groups 1 and 2, n=12 out of 30 patients) would be of great interest and could provide valuable insights.

- Thank you for this suggestion. However, we assessed that the power to performed adequate subgroup analyses was lacking.

Reviewer: 3
Mrs. Rianne Oostenbrink, Erasmus MC - Sophia Children's Hospital
Comments to the Author:
The authors perform a nice study, to validate a large set of CPR on meningitis in a new cohort of patients. Design is appropriate. My main comment, is that although there are no real flaws in the analyses, the message of the paper from the analysis could be much clearer with a change in assessments. It is confusing that the authors mention CNS infections and BM, I assume they only use the BM as outcome for the validation, but it should be confirmed.

- We have adjusted the manuscript in order to present a more clear message about the difference in definition between BM and CNS infections. We indeed use BM as outcome for validation, however we validated the models in our cohort of all children suspected of a CNS infection, since this is the population in which these models will be of use. By validating the models in a more heterogeneous population of all children suspected of a CNS infection, it could give a realistic view of the performance, than for instance comparing performance in only confirmed bacterial versus confirmed viral meningitis cases. Below the changes we made regarding this point of review:

- Page 1, title. We adjusted the title to improve clarity: “Diagnostic prediction models for bacterial meningitis in children with <u>a suspected central nervous system infection</u>: a systematic review and prospective validation study”
- Page 5 introduction, We added a line (underlined) to this sentence: “ Validation of prediction models in a broader population of patients suspected of a central nervous system (CNS) infection is necessary <u>because this is the population in which these models will be of clinical use</u>, …”

Next, they report substantial problems in validation, but conclude that the models show good to excellent test results, so this seems contrasting. I would suggest that the authors assess better and discuss better their observations in the validation and the calibration results from the perspective of models being overfitted, differences in casesmix, and need for recalibration or remodelling. this now is mixed throughout the paper, and reads confusing. authors may consider to come with a message with more solutions

- We adjusted the conclusion of our manuscript because we agree it seems contrasting to report that models show excellent test results but substantial over- and underestimation at the same time.
What we tried to say is that although some of these models provide useful diagnostic information and discrimination can be good, they should not be used as a stand-alone test, and all other information in the clinical setting should be incorporated as well. We have added two lines about this in the concluding paragraph of the discussion.

- We have tried to be more clear about observations in the validation and the calibration results, see below the changed we made regarding this:

- We have added this sentence to the explanatory paragraph in the discussion about differences between our results and previous studies; " This is likely largely due to differences in case-mix between our validation cohort and the original derivation cohorts. "
- We have added to the discussion: " Other common causes of reduced discrimination and calibration are related to methodological problems regarding the algorithm itself, such as statistical overfitting."
- Page 17, we have added the word calibration " To date, a large amount of prediction models for bacterial meningitis have been developed but none showed excellent discrimination <u>or calibration</u> when validated in a broader population of all patients suspected of a CNS infection."

Specific comments:
abstract:
conclusion: impact on patient outcome is a different step in validation of CPRs (See Reilly et al, annals int med 2006), and is not the scope of this manuscript (no studies on impact analyses were included in the review)

We have adjusted the conclusions of our abstract. Thank you for pointing this out.

Methods
p 7 line 5-6 'patients with insufficient amount of data': how many were excluded, and please elaborate on impact of excluding these patients on the result.

-On page 10 we state that in total 468 episodes were included, of which 450 episodes could be used in the analysis. We have added a line about the impact of excluding these patients on the results, in our discussion.

p7 line 8-9: sensitivity analysis should be in analysis/statistic section

-We have moved this sentence to the statistical analysis section.

p7 line 15-20: what is done with the cases of CNS infection, why mentioning this diagnosis separate?

-This line is a description of our cohort.

p8 line 8 'diagnosis of bacterial meningitis based on positive CSF'....: this contrast with your presented definition of BM at page 7, so what definition did you use?

-Thank you for pointing out this textual error. We have corrected this by leaving out 'based on positive CSF culture". We indeed used the previously mentioned definition at page 7, which not only includes patients with a positive CSF culture.

p8 line 40-41: prob< 0.1 low risk, > 0.8 high risk: the predicted risks of a CPR usually do not translate to actual risks perceived by the clinicians on an outcome. It would be more logic to use the threshold as set by the developers of the CPR for decisions in this assessment, rather than an overall prob of <

0.1 an d>0.8. otherwise, show that the thresholds set by the original authors cohere with these probabilities

- What we attempted to show is how often and by how much the risk of bacterial meningitis changed from the a-prior risk, depending on the outcome of the risk score. However, we agree with the reviewer these cut-off values are arbitrary and that pre-defined levels of clinical relevance cannot be stated. We have deleted these analyses from our manuscript.
To give an impression of the change from baseline risk of bacterial meningitis depending on the result (high risk or low risk) from the risk score, we have added supplementary table S3.

p8 line 57-59: excluding a variable from the CPR results by a reduced predicted risk per definition. so this should be corrected if you use subsequently the thresholds of the original authors, and will lead to need of calibration in the large. You could consider to adjust the pred risk by setting a mean value for the missing variable as observed in tht original cohort, thereby correcting the intercept.

-Thank you for this suggestion. We have adjusted the methods and results by stating that if one predictor in the model is missing, the model was still validated, and if 2 or more predictors were missing, this model was excluded for validation. In models in which a continuous variable was missing, the median or mean value in the original cohort was used as observed value in validation in our cohort, and this was reported in the results as well. For models in which a categorical variable was missing (e.g. Gram stain positive or negative) we chose to leave out this variable.
Due to this change, the numbers in the flowchart (total number of articles validated in our cohort) have changed as well (Figure 1).

Results
p9 line 47-50: to assess the power of the developing study cohorts, please report the number of BM cases additional to the cohort size

-This information per study is stated in Table 2.

p 10 line 47-48: please include definitions of the mentioned diagnoses in the paper/methods

-Definitions of diagnoses can be found in the paper mentioned in the methods section, by authors Khatib et al, to which we refer.

p10 line 54: validation cohort includes 30 BM cases. usually it is adviced to include 100 cases and 100 noncases as a minimum for valid validation results. I understand the limitations of having such a cohort with BM. but at least, discuss the impact of this low number of cases on the power of your study, and how the results should be interpreted. this in partciular applies to the sensitivity analyses, where the number of cases are much smaller!

-We agree with this comment and have added more lines in the discussion section on how this might impact the results.

p13 please report the number of BM cases in the two cohorts neonates and >28d given the remark above

-We have added a line at p11, after description of the 3 cohorts, with number of BM cases for each cohort.

discussion
p14 line 53-60 reduced performance is mentioned, but the cohort they are validated in is very different from the original cohorts for some (other ages, other inclusion criteria/severity level). it is logic that this provides a different mean risk, thereby in need for calibration in the large. this does not say any about the performance of the rules, but about the differences in the cohorts

-We agree that difference between our validation cohort and the original derivation cohorts likely explain reduced performance. Our results indicate the performance of prediction models one can expect when applying these rules in a ´real-world´ heterogeneous pediatric practice.

p 15 lines 2-30: this deals with different origin of the populations. so please use in this discussion the results you observed from recalibration. you may add the assessments like Brier to discuss whether indeed differences are related to differences in case-mix versus overestimation in the original cohort. next, please take your small number of cases into account.

-We have added this reason for differences in model performance to this paragraph and added the topic of calibration.

p15 line 32 - 60: the topic machine learning seems a bit out of scope in this paper if there is only one machine learning model in the review?

-We agree that this is a bit of the scope of this review, and have removed this paragraph from the discussion.

p 16 line 45 : lead to difference in performance: see remarks before, this is a clear example that it is needed to explain that this could be corrected by calibration in the large.

-We have added this to the paragraph about differences in model performance, as discussed above

p 16 line 55: low number of cases is a real limitation of the paper, and should be discussed better

-As mentioned at the comment about p10 line 54, we have added more lines in the discussion section on how this might impact the results.

References

1. Geersing GJ, Bouwmeester W, Zuithoff P, Spijker R, Leeflang M, Moons KG. Search filters for finding prognostic and diagnostic prediction studies in Medline to enhance systematic reviews. PLoS One. 2012;7(2):e32844.
2. van Zeggeren IE, Bijlsma MW, Tanck MW, van de Beek D, Brouwer MC. Systematic review and validation of diagnostic prediction models in patients suspected of meningitis. J Infect. 2020;80(2):143-51.

VERSION 2 – REVIEW

**REVIEWER**	Sergeant, Jamie

	University of Manchester, Arthritis Research UK Centre for Epidemiology
**REVIEW RETURNED**	16-Apr-2024

**GENERAL COMMENTS**	The authors have addressed the more straightforward, minor comments that I made in my original review. However, I am still of the opinion that it would be better to have a systematic review and critical appraisal of clinical prediction models in this field as one manuscript, and the external validation of models as a separate manuscript. This would allow each of those elements to be more fully and transparently reported, and help facilitate critical appraisal and evidence synthesis of this work. If the editors are content with a single manuscript that includes both a systematic review and an external validation of models, at the expense of fuller detail, then that is their decision.<br><br>In my previous comments I also noted problems with attempting to validate models for which the full functional form is not available, and with validating models containing variables which are not available in the validation dataset. The authors have taken no action on these points (probably the only action would be to remove these elements). At least the reporting is quite clear though, and anyone appraising this work will be able to see that this is what they have done.<br><br>Finally, the reporting of how missing data is handled could have been further improved. The authors have clarified which variables were used in multiple imputation but not what model was used or its exact parameters. Stating the name of the R package used is not sufficient detail to allow replication of the approach.

**REVIEWER**	Shen, Susanne Dyckhoff<br>LMU Klinikum
**REVIEW RETURNED**	26-Mar-2024

**GENERAL COMMENTS**	All questions that were raised were answered satisfactorily by the authors.

**REVIEWER**	Oostenbrink, Rianne<br>Erasmus MC - Sophia Children's Hospital
**REVIEW RETURNED**	12-Apr-2024

**GENERAL COMMENTS**	Thanks for this revised version of the manuscript. The manuscript is indeed substantially approved, and more clear.<br>My remaining comment is the part on calibration, which could be more discussed in an informative way. What I miss is the discussion of influence of change in incidence of the outcome (calibration of the large), which may be more applicable in the very young, with higher incidence of BM compared to the original populations, and the observation of over/underestimation (calibration slope), possible related to different effects of predictors in different populations. How do these observations of diff in calibration in the large and or calibration slope affect use in practice in new populations? And did the authors observe significant calibration issues (i.e.they report some calibration slopes, but they are with a confidence interval including 1). The statement that some rules resulted in over- and underestimation, should be expanded by discussing how the background of the rules can explain these observations, and adds to the previous

	paper validating rules in adult populations. This applies to both results and discussion sections.

VERSION 2 – AUTHOR RESPONSE

Reviewer: 1

Dr. Jamie Sergeant, University of Manchester, Manchester Academic Health Science Centre

Comments to the Author:

The authors have addressed the more straightforward, minor comments that I made in my original review. However, I am still of the opinion that it would be better to have a systematic review and critical appraisal of clinical prediction models in this field as one manuscript, and the external validation of models as a separate manuscript. This would allow each of those elements to be more fully and transparently reported, and help facilitate critical appraisal and evidence synthesis of this work. If the editors are content with a single manuscript that includes both a systematic review and an external validation of models, at the expense of fuller detail, then that is their decision.

- We agree that multiple manuscripts would be provide us with the opportunity to provide a more detailed discussion. However, our aim was to provide both an overview of all available prediction rules and secondly to compare the performance of these rules in the same external validation set. A manuscript only describing existing prediction models without subsequent validation is of lesser value in our opinion because the reader cannot compare the performance of these individual rules. A manuscript that reports on the external validation of a prediction rule without an overview of all existing prediction models begs the question why those particular prediction models were chosen.

In my previous comments I also noted problems with attempting to validate models for which the full functional form is not available, and with validating models containing variables which are not available in the validation dataset. The authors have taken no action on these points (probably the only action would be to remove these elements). At least the reporting is quite clear though, and anyone appraising this work will be able to see that this is what they have done.

Finally, the reporting of how missing data is handled could have been further improved. The authors have clarified which variables were used in multiple imputation but not what model was used or its exact parameters. Stating the name of the R package used is not sufficient detail to allow replication of the approach.

- We have added a more detailed explanation on how missing data were handled and which imputation model was used in the methods section.

Reviewer: 2

Dr. Susanne Dyckhoff Shen, LMU Klinikum

Comments to the Author:

All questions that were raised were answered satisfactorily by the authors.

Reviewer: 3

Mrs. Rianne Oostenbrink, Erasmus MC - Sophia Children's Hospital

Comments to the Author:

Thanks for this revised version of the manuscript. The manuscript is indeed substantially approved, and more clear.

My remaining comment is the part on calibration, which could be more discussed in an informative way. What I miss is the discussion of influence of change in incidence of the outcome (calibration of the large), which may be more applicable in the very young, with higher incidence of BM compared to the original populations, and the observation of over/underestimation (calibration slope), possible related to different effects of predictors in different populations. How do these observations of diff in calibration in the large and or calibration slope affect use in practice in new populations? And did the authors observe significant calibration issues (i.e. they report some calibration slopes, but they are with a confidence interval including 1). The statement that some rules resulted in over- and underestimation, should be expanded by discussing how the background of the rules can explain these observations, and adds to the previous paper validating rules in adult populations. This applies to both results and discussion sections.

- We have added a more detailed explanation about calibration to the discussion section. We now elaborate more on the poor calibration slopes and calibration in the large, related to the different effect of predictors in different populations as well as the difference in incidence, respectively.

We also expanded our discussion with suggestions for applying these models in clinical practice in new populations.

- Thank you very much for pointing that out. We originally reported both the p-value of the Hosmer-Lemeshow (HL) test, and calibration slope with 95% confidence interval, in the same table. This was clearly confusing. Because calibration slopes are more informative than a single p-value of the HL-test, we decided to only report the calibration slope with 95% confidence interval and the corresponding p-value, and remove the p-value of the HL-test.
